# Comparison of Criteria for Choosing the Number of Classes in Bayesian Finite Mixture Models

**DOI:** 10.1371/journal.pone.0168838

**Published:** 2017-01-12

**Authors:** Kazem Nasserinejad, Joost van Rosmalen, Wim de Kort, Emmanuel Lesaffre

**Affiliations:** 1 Department of Biostatistics, Erasmus MC, Rotterdam, the Netherlands; 2 Department of Hematology, Clinical Trial Center, Erasmus MC Cancer Institute, Rotterdam, the Netherlands; 3 Sanquin Research, Department of Donor Studies, Amsterdam, the Netherlands; 4 Department of Public Health, Academic Medical Center, Amsterdam, the Netherlands; 5 L-Biostat, KU Leuven, Leuven, Belgium; Universitat Wien, AUSTRIA

## Abstract

Identifying the number of classes in Bayesian finite mixture models is a challenging problem. Several criteria have been proposed, such as adaptations of the deviance information criterion, marginal likelihoods, Bayes factors, and reversible jump MCMC techniques. It was recently shown that in overfitted mixture models, the overfitted latent classes will asymptotically become empty under specific conditions for the prior of the class proportions. This result may be used to construct a criterion for finding the true number of latent classes, based on the removal of latent classes that have negligible proportions. Unlike some alternative criteria, this criterion can easily be implemented in complex statistical models such as latent class mixed-effects models and multivariate mixture models using standard Bayesian software. We performed an extensive simulation study to develop practical guidelines to determine the appropriate number of latent classes based on the posterior distribution of the class proportions, and to compare this criterion with alternative criteria. The performance of the proposed criterion is illustrated using a data set of repeatedly measured hemoglobin values of blood donors.

## Introduction

Finite mixture models can be used to capture unobserved heterogeneity in the population by assuming that the population consists of *K* homogeneous subgroups. These models also allow to represent non-standard distributions by an appropriate mixture of standard distributions. However, identifying the number of latent classes (*K*) remains a challenging problem [1–4]. Several criteria exist for choosing the number of latent classes in mixture models in both the frequentist and the Bayesian setting. Whereas information criteria such as the Akaike information criterion (AIC) [5] and the Bayesian information criterion (BIC) [6] seem to be the most popular criteria in a frequentist setting [7–9], no clear consensus on the optimal criterion in a Bayesian setting has yet emerged. Although the deviance information criterion (DIC) [10] is a well-established criterion for comparing different Bayesian models, unfortunately this criterion is not suited to the case of mixture models [7]. Several adaptations of this criterion to mixture models have been proposed [11]. Alternatively, models with different numbers of latent classes can be compared by computing marginal likelihoods, Bayes factors, or by using reversible jump Markov chain Monte Carlo (RJMCMC) techniques [12].

The appropriate number of latent classes is obtained by optimizing one of the criteria by fitting several mixture models with different numbers of classes. However, this procedure is often not easy to apply, as estimating a finite mixture model for different numbers of classes can be time consuming. Furthermore, some of these criteria cannot be calculated using standard software for Bayesian analyses such as WinBUGS, JAGS, or Stan, so that the researcher often has to compute the criteria outside these software packages. RJMCMC sampling is another approach with its own drawbacks. In this algorithm the Markov chain moves between mixture models with different numbers of classes based on carefully selected proposal densities [13, 14]. It can be difficult to derive appropriate proposal densities, especially for complex hierarchical models. Alternative choices such as marginal likelihood approaches, which are generally not available in closed form in mixture models, also yield challenging numerical issues even for mixture models with a moderate number of classes [13].

Rousseau and Mengersen [4] (hereafter R&M) showed that in overfitted mixture models (i.e. a mixture model fitted with more latent classes than present in the data), the superfluous latent classes will asymptotically become empty if the Dirichlet prior on the class proportions is sufficiently vague. Rousseau and Mengersen [4, 15] indicated that their result may lead to a criterion for finding the true number of latent classes by simply excluding latent classes that are negligible in proportion. A subsequent study by Malsiner-Walli et al. [16] proposed a specific implementation of this criterion, and used simulated data to investigate its performance in finding the true number of latent classes. In their implementation, the mixture model is first estimated with a relatively large number of latent classes. The true number of latent classes is then estimated as the mode of the number of non-empty classes, where a class is defined as empty if no subject is assigned to it in a specific MCMC iteration. The advantage of R&M criterion is that it is simple to implement using standard Bayesian software, even for complex statistical models, because the latent class proportions are an automatic byproduct of the estimation.

In this study, we use a criterion that resembles the criterion used by Malsiner-Walli et al. [16]. However, we relax the rather conservative criterion used by Malsiner-Walli et al. [16] that a class is only empty if it contains zero observations, and instead assess the effects of different cut-offs for the proportions in a class. This is more logical, because Rousseau and Mengersen [4] only showed that the class proportions converge to 0 if the sample size approaches infinity, not that they should be 0 with any data set of finite size. The simulation study of Malsiner-Walli et al. only used data sets with well-separated latent classes and did not compare the criterion with alternative methods for choosing the number of latent classes. In our simulation study, we considered various scenarios with different degrees of separation between latent classes as well as longitudinal data, to assess how this criterion performs in a more realistic setting. We also compared the R&M criterion with alternative criteria for estimating the number of latent classes.

We show that both the prior for the class-specific parameters as well as the hyperparameter of the Dirichlet prior distribution for the class proportions have to be chosen carefully to ensure a good performance of this method. We use the simulation results to provide recommended settings, and apply these settings in the analysis of longitudinal hemoglobin (Hb) values of blood donors.

In the next section, background on finite mixture models is presented including a discussion of priors for mixture models. Then methods for choosing the appropriate number of classes in this study are presented. Section ‘simulation studies’ deals with the simulation study in both a univariate and a longitudinal setting. In Section ‘hemoglobin longitudinal data’, a practical example of longitudinal mixture modeling is presented. Finally, the results are discussed and practical recommendations are given in ‘discussion’ section.

## Background on finite mixture models

### Definition of mixture models

A finite mixture model is defined as:

$$\begin{array}{c} f\left(y|\lambda ,\theta ,\gamma \right)=\sum_{j=1}^{K}\lambda_{j}f_{j}\left(y|\theta_{j},\gamma \right), \end{array}$$ (1)

where *f*(*y*|**λ**, *θ*, *γ*) is the density of the observed data, *f*_*j*_(*y*|*θ*_*j*_, *γ*) is the density of the observed data in latent class *j*, *K* is the true number of latent classes and the vector **λ** represents the class proportions, which are non-negative and sum to 1. *θ*_*j*_ is a vector of parameters for the distribution of the data in class *j*, and *γ* is a vector of parameters common to all classes. The observed data *y* can be either univariate or multivariate, and *f*_*j*_(*y*|*θ*, *γ*) may correspond to e.g. a simple Gaussian model or a complex hierarchical model.

Since we use a Bayesian setting, priors need to be chosen for *λ*_*j*_, *θ*_*j*_ (*j* = 1, …, *K*), and *γ*. A challenging issue that arises in Bayesian mixture models is the nonidentifiability of the latent classes. The problem is caused by the invariance of the posterior distribution with respect to permutations of class labeling under symmetric priors and likelihood [14]. This leads to so-called label switching in the MCMC output, and the posterior distributions of class-specific parameters *θ*_*j*_ will be identical and thus useless for inference [17].

### Priors for mixture models

If no relevant prior information for the parameters is available, many researchers prefer to use vague prior distributions whose impact on the posterior distribution of the model parameters is minimal. The most commonly used prior for the class proportions, *λ*_*j*_, is a symmetric Dirichlet distribution, i.e. **λ**|K ∼ Dirichlet(*α*_1_, …, *α_K_*), and *α*_*k*_ = *α* for *k* = 1, …, *K*. Smaller values of *α* correspond with a less informative prior. A flat prior distribution is obtained with *α* = 1, whereas setting *α* = 0 leads to an improper Dirichlet distribution, and also to an improper posterior result.

The choice of *α* is important, as its value can strongly affect the posterior results. Although large values of *α* lead to informative prior distributions, some researchers have suggested to use values larger than 1 (e.g., *α* = 4 or *α* = 10) to avoid solutions with empty classes [18]. When using the marginal likelihood as a criterion (i.e. choosing the number of latent classes that yields the highest value of the marginal likelihood), it has been shown that more informative Dirichlet distributions lead to a lower probability of overestimating the number of latent classes in the data [19, 20].

In contrast, Rousseau and Mengersen [4] have suggested to use smaller values of *α*, with *α* < *d*/2, where *d* is the number of class-specific parameters, i.e. *θ*_*j*_. This recommendation is based on a mathematical proof showing that with a sufficiently vague Dirichlet prior distribution, the proportions of overfitted latent classes will converge to zero as the sample size increases. For *α* greater than *d*/2, the class proportions of overfitted classes will asymptotically converge to nonnegligible values, even if the data are homogeneous. Although the proof given by R&M does not explicitly mention the possibility of parameters that do not vary between classes (i.e. the parameters *γ*), in this paper we will apply the mathematical result also in models with such parameters. However, in all cases the value of *d* is chosen as the dimension of *θ*_*j*_, i.e. the number of class-specific parameters. This is one of the few examples in Bayesian statistics where less informative priors lead to better results [4], as the more informative Dirichlet distributions will overestimate the number of latent classes. Rousseau and Mengersen [4] further argued that with *α* < *d*/2, the posterior distribution of the class proportions has a much more stable behavior than the maximum likelihood estimator. Another disadvantage of using informative Dirichlet priors is that the posterior distributions of the class proportions may be biased, especially in small data sets.

An alternative approach to fixing *α* in advance would be to let the data determine the optimal value for alpha, which means to use a hyperprior specification for *α*, so that *α* is an unknown parameter that is estimated using the data. The prior for *α* could for example be a gamma prior, with *α* ∼ Γ(*ϵ*_1_, *ϵ*_2_) [16, 21], where *ϵ*_1_ and *ϵ*_2_ are the shape and rate parameters of the gamma distribution, respectively.

Priors must also be chosen for the class-specific parameters *θ*_*j*_. In many cases, there is no relevant prior information available for the class-specific parameters, so that the use of vague priors seems appropriate. However, it is generally not possible to use improper priors for the class-specific parameters in finite mixture models, because there is a nonzero posterior probability that at least one of the classes is empty, leading to improper posteriors for the class-specific parameters [22]. Instead one can use minimally informative but diffuse proper priors which lead to diffuse posterior distributions of the class-specific parameters, but the posterior results may be sensitive to the spread of the prior [22].

Data-dependent priors, which are prior distributions that are a function of the observed data, have been proposed instead [3, 22, 23]. Wasserman [22] showed that these prior distributions may have better frequentist properties.

Another approach would be to use a hierarchical prior. For example in a mixture model one can specify a hierarchical prior for the class-specific means as *μ*_*k*_|*b*_0_ ∼ *N*(*b*_0_, *B*_0_), where *b*_0_ ∼ *N*(*m*_0_, *M*_0_). The aim of these hierarchical priors is to minimize the impact of the prior on the posterior. In many finite mixture models the distribution within each class is assumed to be normal, conditional on the observed covariates. Different priors have been proposed in the literature for the class-specific parameters, namely the priors proposed by Nobile et.al [24], and the normal-gamma prior [25] for class-specific means used in Malsiner-Walli et al. [16] combined with the approach of Rousseau and Mengersen [4]. Previous literature showed that the choice of prior has a strong effect on choosing the number of latent classes in mixture models [17].

## Methods for choosing the number of classes

Various approaches have been proposed in the literature for choosing the number of latent classes in mixture models, in both frequentist and Bayesian settings. However, no consensus has emerged regarding which of these methods performs best. In this study we compare a number of well-known Bayesian approaches for choosing the number of latent classes in mixture models. These approaches are described below.

### Deviance information criterion (DIC)

The deviance information criterion (DIC) is a well-known Bayesian criterion for the assessment and comparison of different Bayesian models [10]. The DIC involves a trade-off between goodness of fit (deviance) and model complexity (the effective number of parameters p_D_), and can be calculated as follows:

$$\begin{array}{c} \text{D}(\theta )=-2\log f(y|\theta )+2\log h(y), \end{array}$$

where *h*(*y*) is a standardizing term that is a function of the data alone. Then the estimated effective number of parameters is defined as:

$$\begin{array}{c} {\text{p}}_{\text{D}}=\bar{\text{D}(\theta )}-\text{D}\left(\hat{\theta }\right), \end{array}$$

where $\bar{\text{D}(\theta )}$ is the posterior mean deviance and $\hat{\theta }=E\left[\theta |y\right]$ is the posterior mean of the model parameters. DIC is then defined as:

$$\begin{array}{c} \text{DIC}=-4E_{\theta }\left[\log f\left(y|\theta \right)|y\right]+2\;\log f\left(y|\hat{\theta }\right), \end{array}$$ (2)

$\hat{\theta }=E\left[\theta |y\right]$ ensures that *p*_*D*_ is positive when the density is log-concave in *θ*, but it is not appropriate for discrete parameters *θ* [10, 11]. In mixture models, the parameters *θ* are not identifiable if the prior and likelihood are invariant with respect to the labeling of classes. Therefore, $\hat{\theta }=E\left[\theta |y\right]$ can be a very poor estimator and *p*_*D*_ may become negative [11]. A more relevant choice for $\hat{\theta }$ would be the mode of the posterior distribution [11]. Several adaptations of this criterion were proposed by Celeux et al. for mixture models [11], such as DIC_3_ and DIC_4_. Namely,

$$\begin{array}{c} {\text{DIC}}_{3}=-4E_{\theta }\left[\log f\left(y|\theta \right)|y\right]+2\;\log\hat{f}\left(y\right), \end{array}$$ (3)

where $\hat{f}\left(y\right)=\prod_{i=1}^{n}\hat{f}\left(y_{i}\right)$, $\hat{f}\left(y_{i}\right)=\frac{1}{M}\sum_{m=1}^{M}\sum_{j=1}^{K}\lambda_{j}^{m}f_{j}\left(y|\theta_{j}^{m}\right)$, *M* denotes the number of MCMC iterations, $\lambda_{j}^{m}$ and $\theta_{j}^{m}$ are the results of the *m*th MCMC iteration, and

$$\begin{array}{c} {\text{DIC}}_{4}=-4E_{\theta ,Z}\left[\log f\left(y,Z|\theta \right)|y\right]+2E_{Z}\left[\log f(y,Z\right|E_{\theta }\left[\theta |y,Z\right]\left|y\right], \end{array}$$ (4)

where *Z* = (*z*_1_, …, *z*_*n*_) is the class assignment vector of observations (individuals). To compute DIC_4_, it is necessary to calculate the posterior expectation for each possible value of *z*. Among various DICs studied by Celeux et al., these two DICs were found to be the most reliable criteria by the authors [11].

### Reversible jump MCMC algorithm

Another fully Bayesian approach is the reversible jump MCMC algorithm (RJMCMC), as introduced by Richardson and Green [12], which is an extension of the standard MCMC. RJMCMC allows sampling of the posterior distribution on spaces of varying dimensions. In this algorithm the Markov chain moves between finite mixture models with different number of classes based on carefully selected degenerated proposal densities, but which are in general not easy to design [14, 26].

### Rousseau and Mengersen’s criterion

Rousseau and Mengersen (R&M) [4] proved that the posterior behavior of an overfitted mixture model depends on the chosen prior on the proportions *λ*_*j*_. They showed that an overfitted mixture model converges to the true mixture, if the Dirichlet-parameters *α*_*j*_ of the prior are smaller than *d*/2 (*d* is the dimension of the class-specific parameters). This result can be used to define a criterion for choosing the true number of latent classes in a mixture model. Basically, a deliberately overfitted mixture model with *K*_max_ (*K*_max_ > *K*) latent classes is fitted to the data. A sparse prior (Dirichlet distribution with *α*_*j*_ < d/2) on the proportions is then assumed to empty the superfluous classes (*K*_max_ − *K*) during MCMC sampling.

Various criteria can be used for a class to be declared empty. For instance, one could declare a class empty if the number of observations assigned to that class is smaller than a certain proportion of the observations in the data set (e.g. *ψ*). In other words, the (assumed) true number of non-empty classes (*K*) could be computed in each MCMC iteration as:

$$\begin{array}{c} K^{\left(m\right)}=K_{\text{max}}-\sum_{j=1}^{K_{\text{max}}}I\left\{\frac{N_{j}^{\left(m\right)}}{N}\leq \psi \right\}, \end{array}$$ (5)

where *K*^(*m*)^ is the number of non-empty classes in iteration *m* of MCMC sampling, $N_{j}^{\left(m\right)}$ is the number of observations allocated to class *j* at iteration *m*, *N* is the total number of observations and *I* denotes the indicator function. *ψ* can be set to a predefined value, e.g. 0, 0.01, 0.02, or 0.05. Then one can derive the number of non-empty classes based on the posterior mode of the number of non-empty classes based on all MCMC iterations.

### Bayesian information criterion

The Bayesian information criterion (BIC) [6] is a well-known frequentist criterion, which has been shown to be consistent for choosing the number of latent classes in mixture models [9]. BIC is defined as follows:

$$\begin{array}{c} \text{BIC}=-2\left[\log f\left(y|\hat{\theta }\right)\right]+g\;\log\left(n\right), \end{array}$$ (6)

where $\hat{\theta }$ is the maximum-likelihood estimate of the parameter *θ*, *g* is the number of free parameters in the model, and *n* is the number of observations in the data.

## Simulation studies

To investigate the performance of the criterion proposed by R&M compared to other well-known approaches, we set up two simulation studies with different scenarios. The first simulation study is based on one-dimensional data, whereas the second simulation study uses longitudinal data.

### Simulation study A: univariate Gaussian mixture

In this simulation study, we consider a univariate Gaussian mixture, i.e. a location-scale mixture of univariate normal distributions:

$$\begin{array}{c} f\left(y_{i}|\lambda ,\mu ,\sigma^{2}\right)=\sum_{j=1}^{K}\lambda_{j}N\left(y_{i}|\mu_{j},\sigma_{j}^{2}\right), \end{array}$$ (7)

where *f*(*y*_*i*_|**λ**, *μ*, *σ*^2^) is the density of the observed data *y*_*i*_ (*i* = 1, …, *n*), *n* is the number of independent observations, $N(y_{i}|\mu_{j},\sigma_{j}^{2})$ is the density of the normal distribution with mean *μ*_*j*_ and variance $\sigma_{j}^{2}$, *K* is the true number of latent classes and *λ*_*j*_ is the proportion of latent class *j*.

**Fig 1:**
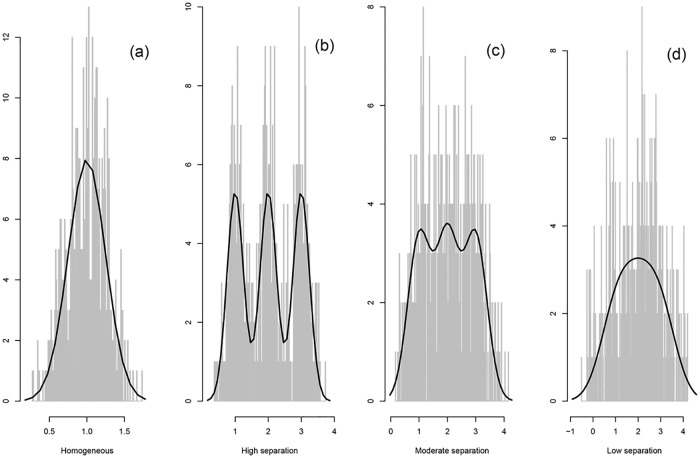
Univariate simulated data study. Histograms of randomly selected generated data sets. The solid lines represent the true marginal densities.

We simulate data from this model using *n* = 500 observations with different numbers of latent classes, and different degrees of separation (i.e. “low”, “moderate”, and “high” separation). Our definition of “low”, “moderate”, and “high” separation is somewhat subjective, and is based on the percentage of variation in the data that can be explained by the clustering structure, i.e. $\sigma_{E(Y|Z)}^{2}/\sigma_{Y}^{2}$ where $\sigma_{Y}^{2}$ denotes the marginal variance of the data, *Z* is an indicator variable for the latent class, and $\sigma_{E(Y|Z)}^{2}$ is the between-class variance. We also assessed the degree of separation between latent classes using the overlapping coefficient (OVL), which is the area under the probability density functions simultaneously [27]. The following four scenarios were considered:

- **Scenario A1:** No clustering structure: *K* = 1 class with *μ*_1_ = 1 and *σ*_1_ = 0.25, see Fig 1(a).
- **Scenario A2:** High separation ($\sigma_{E(Y|Z)}^{2}/\sigma_{Y}^{2}=0.80$, OVL = 0.06): *K* = 3 classes with *μ*_1_ = 1, *μ*_2_ = 2, *μ*_3_ = 3, and *σ*_1_ = *σ*_2_ = *σ*_3_ = 0.25, see Fig 1(b).
- **Scenario A3:** Moderate separation ($\sigma_{E(Y|Z)}^{2}/\sigma_{Y}^{2}=0.70$, OVL = 0.29): *K* = 3 classes with *μ*_1_ = 1, *μ*_2_ = 2, *μ*_3_ = 3, and *σ*_1_ = *σ*_2_ = *σ*_3_ = 0.4, see Fig 1(c).
- **Scenario A4:** Low separation ($\sigma_{E(Y|Z)}^{2}/\sigma_{Y}^{2}=0.60$, OVL = 0.74): *K* = 3 classes with *μ*_1_ = 1, *μ*_2_ = 2, *μ*_3_ = 3, and *σ*_1_ = *σ*_2_ = *σ*_3_ = 0.7, see Fig 1(d).

In the base-case analysis, the data are simulated using equal class proportions (i.e. *λ*_*j*_ = 1/*K* for each class *j*). The histograms of these simulated data for a randomly selected data set are displayed in Fig 1, together with the true marginal densities. Separation decreases from Fig 1(b) to 1(d), to end in a unimodal distribution.

We implemented the criterion proposed by R&M, and we compared this criterion with the results of RJMCMC [12], DIC_3_ and DIC_4_ [11], and BIC. To establish whether a class is empty under the R&M criterion we used different values for the cut-off (*ψ*) i.e., 0, 0.01, 0.02, and 0.05 of observations in the sample, and the maximum number of latent classes was set to *K*_max_ = 10.

The prior for the class proportions *λ* was chosen to be a symmetric Dirichlet distribution with hyper-parameter equal to *α* = 0.00001, 0.001, 0.01, 0.05, 0.1, 0.3, 0.5, 0.9.

For the priors of the class-specific means, we considered both a normal-gamma prior and a vague prior. The vague prior was *μ_j_* ∼ *N*(0, 1000). The normal-gamma prior is a hierarchical data-dependent prior that places a normal prior on the prior mean and a shrinkage prior on the prior variance [16]. This prior for a univariate mixture model can be defined as follows:

$$\begin{array}{c} \mu_{k}|\lambda \;,b_{0}∼N\left(b_{0},\eta \;R^{2}\right), \end{array}$$

where *η* ∼ Γ(*ν*_1_, *ν*_2_) and *b*_0_ ∼ *N*(*m*_0_, *M*_0_), *m*_0_ and *R* are the median and range of the data, respectively. $M_{0}^{-1}$ is set to 0 (since this is not possible in practice here we set $M_{0}^{-1}=10^{-7}$). The hyper-parameters *ν*_1_ and *ν*_2_ are set to 0.5 to allow considerable shrinkage of the prior variance of class means [16].

For the priors of the class-specific variance, we also considered a hierarchical data-dependent prior and a vague prior. The hierarchical data-dependent prior on the class-specific variances was implemented by Malsiner-Walli et al. [16] in a multivariate mixture model, and is given by:

$$\begin{array}{c} 1/\sigma_{k}^{2}∼\Gamma \left(\beta_{1}=1.25,\beta_{2}=1/\left(2C_{0}\right)\right), \end{array}$$

where *C*_0_ ∼ Γ(*ϵ*_1_ = 0.25, *ϵ*_2_ = 20/*R*^2^). The vague prior on the class-specific variances was $\sigma_{j}^{2}\overset{}{∼}U\left(0,10\right)$.

We used a full factorial design to vary a) the number of latent classes and the degree of separation (using the four scenarios described above), b) the criterion for determining the number of latent classes (i.e. the R&M criterion with different cut-off values, RJMCMC, DIC_3_, DIC_4_, and BIC), and c) the value of *α* in the Dirichlet distribution (i.e. *α* = 0.00001, 0.001, 0.01, 0.05, 0.1, 0.3, 0.5, or 0.9).

Three additional factors were varied in sensitivity analyses. In these sensitivity analyses, only the scenario with high separation between classes was simulated, but the other factors in the full factorial design were not fixed.

Two sensitivity analyses consisted of a) changing the sample size of the data set (i.e. to 100 and 1000 observations) and b) simulating data with unequal proportions of the latent classes, including one small class, using *λ*_1_ = 0.475, *λ*_2_ = 0.475, *λ*_3_ = 0.05. Furthermore, we investigated the sensitivity of the criteria to outlying values, by running Scenario A1 with two extreme values added at each tail of the distribution. Finally, we also performed a sensitivity analysis for the number of latent classes, with *K* ranging from *K* = 1 to *K* = 6, with *n* = 100 × *K* and means chosen as *μ*_*j*_ = *j* for *j* = 1, …, *K* and *σ*_*j*_ = 0.25 and also *σ*_*j*_ = 0.40.

We generated 50 data sets for each setting in the base-case analysis and the sensitivity analyses, except for the sensitivity analyses with varying number of classes, which used only 20 data sets. The low number of simulated data sets for these sensitivity analyses was necessary to limit the total computation time. MCMC sampling is run for each data set for 50,000 iterations after discarding the first 5,000 iterations (burn-in). Computations were performed using the following packages in R: rjags for the R&M criterion (see S1 and S2 Figs in Supplementary Material Section), Rmixmod and lcmm for calculating BIC in a frequentist setting, and mixAK for the RJMCMC technique. To be able to compute DIC_3_ and DIC_4_, an MCMC sampler for the model parameters and the class assignments in the univariate mixture model was programmed in R. The programs of the simulation studies can be obtained by contacting the corresponding author.

### Simulation study A: results

Table 1 shows the simulation results of Scenario A1. This table presents the success rate (the percentage of data sets in which the true number of clusters was obtained) of the different approaches, the mode of the estimated number of classes is presented in parentheses. The criterion of Rousseau and Mengersen is denoted as R&M^NG^ if hierarchical priors are used for both the class-specific mean and the class-specific variance, and as R&M^NI^ if the vague priors are used for both the class-specific mean and the class-specific variance, with the cut-off value for defining a class to be empty as a subscript. For example, ${\text{R\&M}}_{0.02}^{\text{NI}}$ represents the Rousseau and Mengersen criterion with the vague priors for both the class-specific mean and the class-specific variance where *ψ* = 0.02.

**Table 1:** The results of Scenario A1. Percentage of data sets in which the true number of clusters was found, with the mode of the estimated number of classes in parentheses.

*α*	RJMCMC	${\text{R\&M}}_{0}^{\text{NG}}$	${\text{R\&M}}_{0.01}^{\text{NG}}$	${\text{R\&M}}_{0.02}^{\text{NG}}$	${\text{R\&M}}_{0.05}^{\text{NG}}$	${\text{R\&M}}_{0}^{\text{NI}}$	${\text{R\&M}}_{0.01}^{\text{NI}}$	${\text{R\&M}}_{0.02}^{\text{NI}}$	${\text{R\&M}}_{0.05}^{\text{NI}}$	DIC_3_	DIC_4_
0.00001	—	100%(1)	100%(1)	100%(1)	100%(1)	100%(1)	100%(1)	100%(1)	100%(1)	0%(5)	0%(5)
0.001	18%(10)	100%(1)	100%(1)	100%(1)	100%(1)	100%(1)	100%(1)	100%(1)	100%(1)	0%(3)	0%(3)
0.01	28%(1)	98%(1)	98%(1)	98%(1)	98%(1)	100%(1)	100%(1)	100%(1)	100%(1)	100%(1)	20%(5)
0.05	90%(1)	22%(2)	80%(1)	84%(1)	92%(1)	100%(1)	100%(1)	100%(1)	100%(1)	100%(1)	72%(1)
0.1	98%(1)	2%(4)	10%(3)	18%(2)	40%(2)	100%(1)	100%(1)	100%(1)	100%(1)	100%(1)	100%(1)
0.3	98%(1)	0%(8)	0%(6)	0%(5)	0%(3)	98%(1)	100%(1)	100%(1)	100%(1)	98%(1)	100%(1)
0.5	98%(1)	0%(9)	0%(7)	0%(6)	0%(5)	96%(1)	98%(1)	98%(1)	100%(1)	96%(1)	100%(1)
0.9	98%(1)	0%(10)	0%(9)	0%(8)	0%(6)	96%(1)	98%(1)	98%(1)	100%(1)	94%(1)	100%(1)

The success rate of BIC using a frequentist approach was 100%.

In this scenario, the models cannot underestimate the number of classes. Small values for *α* for both a normal-gamma prior and a vague prior in the R&M criterion result in a better estimation of the true number of latent classes. However, the R&M criterion with a normal-gamma prior requires much lower values of *α* (i.e. *α* < 0.1) to obtain adequate results compared to the R&M criterion with the vague prior, in which any value of *α* below 0.5 leads to good results. The other approaches (i.e. RJMCMC, DIC_3_, and DIC_4_) show better results with larger values for *α*. In case of a very low value of *α*, the convergence of the MCMC sampler in the RJMCMC method was poor and therefore no results are reported in the tables for this method with *α* = 0.00001.

Table 2 shows the simulation results of Scenario A2 (high separation), Scenario A3 (moderate separation), and Scenario A4 (low separation). In Scenario A2, small values for *α* (i.e. *α* < 0.05) in the R&M criterion result in a perfect estimation of the true number of latent classes. The number of classes is overestimated by the R&M criterion with the normal-gamma prior for higher values of *α*. No such overestimation is observed for the vague prior. Similar results were obtained in the sensitivity analysis for the number of latent classes (see S1 and S2 Tables). In that sensitivity analysis, the normal-gamma prior yielded good results with values of *α* < 0.1, but the vague only gave good results for larger values of *α*, with *α* > 0.05. RJMCMC and DIC_3_ gave the best results with larger values for *α* (*α* > 0.1). The performance of DIC_4_ does not seem to depend on the value of *α*, but it is not very good, with the probability of finding the true number of latent classes ranging from 50 to 70%. In the sensitivity analysis for the sample size, the number of classes is underestimated in case a low value of *α* is used with a small sample size of 100 observations, but it is estimated accurately in the other situations (see S3 Table).

**Table 2:** The results of Scenario A2–A4. Percentage of data sets in which the true number of clusters was found, with the mode of the estimated number of classes in parentheses.

Scenario	*α*	RJMCMC	${\text{R\&M}}_{0}^{\text{NG}}$	${\text{R\&M}}_{0.01}^{\text{NG}}$	${\text{R\&M}}_{0.02}^{\text{NG}}$	${\text{R\&M}}_{0.05}^{\text{NG}}$	${\text{R\&M}}_{0}^{\text{NI}}$	${\text{R\&M}}_{0.01}^{\text{NI}}$	${\text{R\&M}}_{0.02}^{\text{NI}}$	${\text{R\&M}}_{0.05}^{\text{NI}}$	DIC_3_	DIC_4_
Scenario A2 (high separation)	0.00001	—	100%(3)	100%(3)	100%(3)	100%(3)	100%(3)	100%(3)	100%(3)	100%(3)	8%(5)	68%(3)
	0.001	6%(8)	100%(3)	100%(3)	100%(3)	100%(3)	100%(3)	100%(3)	100%(3)	100%(3)	6%(5)	68%(3)
	0.01	16%(4)	100%(3)	100%(3)	100%(3)	100%(3)	100%(3)	100%(3)	100%(3)	100%(3)	24%(5)	54%(3)
	0.05	54%(3)	0%(4)	84%(3)	98%(3)	100%(3)	100%(3)	100%(3)	100%(3)	100%(3)	94%(3)	52%(3)
	0.1	94%(3)	0%(5)	0%(4)	12%(4)	86%(3)	100%(3)	100%(3)	100%(3)	100%(3)	100%(3)	68%(3)
	0.3	100%(3)	0%(8)	0%(6)	0%(5)	0%(4)	100%(3)	100%(3)	100%(3)	100%(3)	100%(3)	50%(3)
	0.5	100%(3)	0%(9)	0%(8)	0%(6)	0%(5)	98%(3)	100%(3)	100%(3)	100%(3)	100%(3)	64%(3)
	0.9	100%(3)	0%(10)	0%(9)	0%(8)	0%(6)	6%(4)	80%(3)	92%(3)	98%(3)	100%(3)	94%(3)
Scenario A3 (moderate separation)	0.00001	—	4%(2)	4%(2)	4%(2)	4%(2)	0%(2)	0%(2)	0%(2)	0%(2)	20%(5)	10%(1)
	0.001	10%(10)	6%(2)	6%(2)	6%(2)	6%(2)	2%(2)	2%(2)	2%(2)	2%(2)	18%(5)	8%(1)
	0.01	16%(2)	36%(2)	34%(2)	34%(2)	34%(2)	2%(2)	2%(2)	2%(2)	2%(2)	46%(3)	6%(1)
	0.05	2%(2)	38%(4)	88%(3)	86%(3)	74%(3)	2%(2)	2%(2)	2%(2)	2%(2)	28%(2)	8%(1)
	0.1	4%(2)	0%(5)	4%(4)	32%(3)	94%(3)	4%(2)	4%(2)	2%(2)	2%(2)	8%(2)	8%(1)
	0.3	6%(2)	0%(8)	0%(6)	0%(4)	0%(4)	28%(2)	28%(2)	28%(2)	28%(2)	2%(2)	4%(2)
	0.5	8%(2)	0%(9)	0%(8)	0%(7)	0%(5)	60%(3)	48%(2)	46%(2)	46%(2)	4%(2)	4%(5)
	0.9	10%(2)	0%(10)	0%(9)	0%(8)	0%(6)	0%(4)	50%(3)	66%(3)	94%(3)	10%(2)	8%(2)
Scenario A3 (low separation)	0.00001	—	0%(1)	0%(1)	0%(1)	0%(1)	0%(1)	0%(1)	0%(1)	0%(1)	0%(5)	8%(5)
	0.001	8%(1)	0%(1)	0%(1)	0%(1)	0%(1)	0%(1)	0%(1)	0%(1)	0%(1)	78%(3)	46%(3)
	0.01	6%(1)	0%(1)	0%(1)	0%(1)	0%(1)	0%(1)	0%(1)	0%(1)	0%(1)	4%(1)	34%(3)
	0.05	0%(1)	46%(3)	2%(2)	0%(2)	0%(2)	0%(1)	0%(1)	0%(1)	0%(1)	4%(1)	0%(1)
	0.1	0%(1)	2%(4)	80%(3)	64%(3)	16%(2)	0%(1)	0%(1)	0%(1)	0%(1)	6%(1)	0%(1)
	0.3	0%(1)	0%(8)	0%(6)	0%(5)	8%(4)	0%(2)	0%(1)	0%(1)	0%(1)	10%(1)	2%(1)
	0.5	0%(1)	0%(9)	0%(7)	0%(6)	0%(5)	62%(3)	0%(2)	0%(2)	0%(2)	14%(1)	0%(1)
	0.9	0%(1)	0%(10)	0%(9)	0%(8)	0%(6)	0%(5)	6%(4)	44%(4)	98%(3)	12%(2)	0%(1)

The success rates of BIC using a frequentist approach for high, moderate, and low levels of separation were 100%(3), 16%(2), and 0%(1), respectively.

When looking at Scenario A3 (moderate separation) and Scenario A4 (low separation), a different picture emerges. These results show that the R&M criterion may underestimate the true number of latent classes for low values of *α*. Namely, the R&M criterion with the normal-gamma prior underestimates the number of classes with low values of *α* and overestimates this number with high values of *α*. There is a narrow range around values of *α* = 0.05 in which the performance of this criterion is good, and this range seems to depend on the cut-off for defining a class to be empty. On the other hand, the R&M criterion with the vague prior almost always underestimates the number of latent classes in Scenario A3 and Scenario A4. Underestimation rarely occurs with higher values of *α*, but a large value for *α* may result in overestimating the true number of latent classes. In Scenario A4, in which the distribution of the data looks unimodal, all approaches except ${\text{R\&M}}_{0.02}^{\text{NI}}$ and ${\text{R\&M}}_{0.05}^{\text{NI}}$ perform poorly, and most methods detect only a single class.

As a sensitivity analysis, we simulated a heterogeneous population with three unequal proportions, i.e. *λ*_1_ = 0.475, *λ*_2_ = 0.475, *λ*_3_ = 0.05, *μ*_1_ = 1, *μ*_2_ = 2, *μ*_3_ = 3 and *σ*_1_ = *σ*_2_ = *σ*_3_ = 0.25 (high separation), see Table 3 for the results. Here we performed the R&M criterion only with the vague prior. These results are consistent with the results of Scenario A2. The performance of the R&M criterion is quite good except for ${\text{R\&M}}_{0.05}^{\text{NI}}$ since the smallest class proportion is 5%, the cut-off defined for a class to be empty. Finally, in case a few outlying values were added to the homogeneous data of Scenario A1, the outlying values were assigned to different classes when the cut-off *ψ* was lower than 0.02 (see S4 Table).

**Table 3:** Unequal proportions heterogeneous scenario; a heterogeneous population with three clusters. *λ*_1_ = 0.475, *λ*_2_ = 0.475, *λ*_3_ = 0.05, *μ*_1_ = 1, *μ*_2_ = 2, *μ*_3_ = 3 and *σ*_1_ = *σ*_2_ = *σ*_3_ = 0.25 (high separation). Percentage of data sets in which the true number of clusters was found, with the mode of the estimated number of classes in parentheses.

*α*	RJMCMC	${\text{R\&M}}_{0}^{\text{NI}}$	${\text{R\&M}}_{0.01}^{\text{NI}}$	${\text{R\&M}}_{0.02}^{\text{NI}}$	${\text{R\&M}}_{0.05}^{\text{NI}}$	DIC_3_	DIC_4_
0.00001	—	100%(3)	100%(3)	100%(3)	48%(2)	0%(5)	14%(4)
0.001	6%(7)	98%(3)	98%(3)	98%(3)	48%(2)	2%(5)	14%(4)
0.01	36%(4)	98%(3)	98%(3)	98%(3)	50%(3)	40%(3)	24%(4)
0.05	70%(3)	100%(3)	100%(3)	100%(3)	50%(3)	78%(3)	16%(4)
0.1	98%(3)	100%(3)	100%(3)	100%(3)	50%(3)	98%(3)	20%(4)
0.3	100%(3)	100%(3)	100%(3)	100%(3)	54%(3)	98%(3)	28%(4)
0.5	100%(3)	100%(3)	100%(3)	100%(3)	56%(3)	100%(3)	44%(3)
0.9	100%(3)	20%(4)	88%(3)	96%(3)	80%(3)	100%(3)	92%(3)

The success rate of BIC using a frequentist approach was 98%(3).

### Simulation study B: a longitudinal study with a mixture of Gaussian random effects distributions

Simulation study A enabled us to compare different criteria in a simple setting. However, mixtures also appear in more complicated models, where it may be difficult to calculate some of the criteria that were evaluated in Simulation study A. However, the calculation of the R&M criterion should still be feasible in that case. To verify the performance of the R&M criterion we tested its performance based on a simulation study for a mixture model with longitudinal data.

In this simulation study we generate data from a growth mixture model, which is also known as a latent class mixed effects model [28, 29] with a mixture model on the random effects [29]. The density function in a Gaussian growth mixture model can be expressed as Eq 1, where *f*_*j*_(*y*) is the density function that describes the trajectory for class *j*. The vector *θ*_*j*_ represents the parameters that are associated with the trajectory of class *j*. The growth mixture model for individuals that belong to latent class *j* can be expressed as:

$$\begin{array}{c} y_{it|j}=\theta_{j0}+b_{ij0}+\left(\theta_{j1}+b_{ij1}\right)\;{\text{time}}_{it}+\epsilon_{it}, \end{array}$$

where *y*_*it*|*j*_ is the *t*th observation of the *i*th individual, given that this individual is in latent class *j*, respectively. *θ*_*j*0_ and *θ*_*j*1_ are the fixed intercept and slope of the *j*th latent class. *b*_*ij*0_ and *b*_*ij*1_ are the random intercept and slope of the *j*th latent class that are assumed to be bivariate normally distributed with mean zero and a class-specific variance-covariance structure. The residuals *ϵ*_*it*_ are now assumed to be normally distributed, and independent of the random effects. Thus in this model the class-specific parameters (i.e. *θ*_*j*_ in Eq 1) consist of the fixed intercept and slopes and the variances and covariances of the random effects; the parameters common to all classes (i.e. *γ* in Eq 1) consist only of the variance of *ϵ*_*it*_.

In this simulation study we computed the R&M criterion and the BIC. To establish whether a class is empty with the R&M criterion we used different values for the cut-off (*ψ*) as in Section ‘simulation study A’ (i.e., 0, 0.01, 0.02, and 0.05 of observations in the sample), and the maximum number of latent classes was also set to *K*_max_ = 10. Here we considered a homogeneous population (*K* = 1) and a heterogeneous population (*K* = 3).

**Fig 2:**
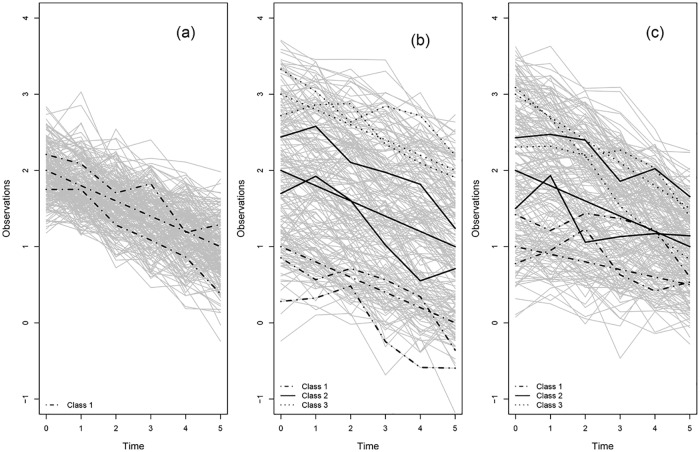
Longitudinal simulated data study. The left profile belongs to a homogeneous population with one class. The middle one belongs to a population with three classes where classes differ only in intercept, and the right profile belongs to a heterogeneous population with three classes where classes differ both in intercept and slope.

- **Scenario B1:** (homogeneous data with a random intercept and slope): *K* = 1 class with *θ*_10_ = 2 and *θ*_11_ = −0.2, **b**_**i1**_ ∼ *N*_2_(**0**, **Σ**), $\Sigma =[\begin{array}{cc} 0.25^{2} & 0\\ 0 & 0.025^{2} \end{array}]$ and the residuals *ϵ*_*it*_ are normally distributed with variance of 0.25^2^, *ϵ_it_* ∼ *N*(0, 0.25^2^), and independent of the random effects, see Fig 2(a). The data were generated as
  $$\begin{array}{c} y_{it|j}=\theta_{j0}+b_{ij0}+\left(\theta_{j1}+b_{ij1}\right)\;{\text{time}}_{it}+\epsilon_{it}, \end{array}$$
  and this model was also used for the analysis, with an unstructured random effects variance-covariance matrix. The class-specific parameters thus consisted of the fixed intercept *θ*_*j*0_ and slope *θ*_*j*1_, as well as 3 parameters for *Σ*, so that d = 5.
- **Scenario B2:** (heterogeneous data with a random intercept): *K* = 3 classes with *θ*_10_ = 1, *θ*_20_ = 2, *θ*_30_ = 3 and *β* = −0.2, *b*_*ij*0_ ∼ *N*(0, 0.25^2^), *b*_*ij*1_ = 0 (a random intercept model) for *j* = 1, 2, 3 and the residuals *ϵ*_*it*_ are normally distributed with variance of 0.25^2^, i.e., *ϵ_it_* ∼ *N*(0, 0.25^2^), and independent of the random effects, see Fig 2(b). The data were generated as
  $$\begin{array}{c} y_{it|j}=\theta_{j0}+b_{ij0}+\beta \;{\text{time}}_{it}+\epsilon_{it}, \end{array}$$
  and this model was also used for the analysis. The class-specific parameters thus consisted of the fixed intercept and the variance of the random intercept, so that d = 2.
- **Scenario B3:** (heterogeneous data with a random intercept and slope): *K* = 3 classes with *θ*_10_ = 1, *θ*_20_ = 2, *θ*_30_ = 3 and *θ*_11_ = −0.1, *θ*_21_ = −0.2, and *θ*_31_ = −0.3, *b*_*ij*0_ ∼ *N*(0, 0.25^2^), **b_ij_** ∼ *N*_2_(**0**, **Σ**), $\Sigma =[\begin{array}{cc} 0.25^{2} & 0\\ 0 & 0.025^{2} \end{array}]$ for *j* = 1, 2, 3 and the residuals *ϵ*_*it*_ are normally distributed with variance of 0.25^2^, *ϵ_it_* ∼ *N*(0, 0.25^2^), and independent of the random effects, see Fig 2(c). The data were generated as
  $$\begin{array}{c} y_{it|j}=\theta_{j0}+b_{ij0}+\left(\theta_{j1}+b_{ij1}\right)\;{\text{time}}_{it}+\epsilon_{it}, \end{array}$$
  and this model was also used for the analysis, with an unstructured random effects variance-covariance matrix. The class-specific parameters thus consisted of the fixed intercept *θ*_*j*0_ and slope *θ*_*j*1_, as well as 3 parameters for *Σ*, so that d = 5.

Vague priors were specified for the class-specific parameters *θ*_*j*0_ and *θ*_*j*1_, i.e. *N*(0, 10^3^). An Γ^−1^(10^−3^, 10^−3^) was specified for the variance of the residuals (this prior also used for the variance of random intercept in Scenario B2). An Inv-Wishart(R, df) distribution was specified for the variance-covariance structure of the random intercept and random slope. We set the degrees of freedom, df, to 3 and the scale parameter matrix, R, to a diagonal matrix with small values, i.e. 10^−3^ [30]. For the class membership probability a Dirichlet distribution with different values (i.e., *α* = 0.00001, 0.001, 0.01, 0.05, 0.1, 0.3, 0.5, 1.0, 1.5, 2.0, and 2.5, where *K*_max_ = 10) for the class proportions was specified. Larger values here were specified since in Scenario B1 and Scenario B3 *d* = 5.

In this analysis, the data are simulated using equal class proportions (i.e. *λ*_*j*_ = 1/*K* for each class *j*). Fig 2 shows a randomly selected generated data set for the three scenarios.

In a sensitivity analysis, we also fitted the random intercept and slope model to the data of Scenario B2 (which were generated with only a random intercept). The purpose of this analysis was to investigate the performance of the criteria in case the statistical model does not match exactly with how the data were generated.

We generated 50 data sets for each setting consisting of 200 subjects and 6 observations per subject. MCMC sampling is run for each data set for 50,000 iterations after discarding the first 5,000 iterations (burn-in).

### Simulation study B: results

The simulation results of Scenario B1 (homogeneous data with a random intercept and slope) show that the R&M criterion with the vague prior estimates the true number of classes perfectly. The results of this simulation are presented in S5 Table in Supplementary Material Section.

Table 4 shows the simulation results of Scenario B2 (heterogeneous data with a random intercept). In this scenario *d* = 2, therefore *α* should be smaller than 1 to make sure that overfitted classes become empty asymptotically [4]. In this scenario, large values for *α* (i.e. 0.1 < *α* < 0.9) in the R&M^NI^ criterion result in an accurate estimation of the true number of latent classes. An underestimation of the number of classes is observed for the R&M^NI^ criterion when a lower value of *α* is used. In this scenario, different cut-offs lead to the same results.

**Table 4:** The results of Scenario B2. Percentage of data sets in which the true number of clusters was found, with the mode of the estimated number of classes in parentheses.

*α*	${\text{R\&M}}_{0}^{\text{NI}}$	${\text{R\&M}}_{0.01}^{\text{NI}}$	${\text{R\&M}}_{0.02}^{\text{NI}}$	${\text{R\&M}}_{0.05}^{\text{NI}}$
0.00001	4%(1)	4%(1)	4%(1)	4%(1)
0.001	4%(2)	4%(2)	4%(2)	4%(2)
0.01	18%(2)	18%(2)	18%(2)	18%(2)
0.05	48%(2)	48%(2)	48%(2)	48%(2)
0.1	74%(3)	74%(3)	74%(3)	74%(3)
0.3	90%(3)	90%(3)	90%(3)	90%(3)
0.5	96%(3)	98%(3)	98%(3)	100%(3)
0.9	8%(4)	14%(4)	20%(4)	34%(4)

The success rate of BIC using a frequentist approach was 98(3)%.

Table 5 shows the simulation results of Scenario B3 (heterogeneous data with a random intercept and slope). In this scenario *d* = 5, therefore *α* should be smaller than 2.5. In this scenario, setting *α* = 2.0 in the R&M^NI^ criterion yields the most precise estimation of the true number of latent classes. Using this value for *α*, the result of the R&M criterion was better than BIC. An underestimation of the number of classes is observed for the R&M^NI^ criterion when a lower value of *α* is used. Larger values for *α* lead to an overestimation of the true number of latent classes.

**Table 5:** The results of Scenario B3. Percentage of data sets in which the true number of clusters was found, with the mode of the estimated number of classes in parentheses.

*α*	${\text{R\&M}}_{0}^{\text{NI}}$	${\text{R\&M}}_{0.01}^{\text{NI}}$	${\text{R\&M}}_{0.02}^{\text{NI}}$	${\text{R\&M}}_{0.05}^{\text{NI}}$
0.00001	4%(2)	4%(2)	4%(2)	4%(2)
0.001	4%(2)	4%(2)	4%(2)	4%(2)
0.01	6%(2)	6%(2)	6%(2)	6%(2)
0.05	6%(2)	6%(2)	6%(2)	6%(2)
0.1	6%(2)	6%(2)	6%(2)	6%(2)
0.3	10%(2)	10%(2)	10%(2)	10%(2)
0.5	10%(2)	10%(2)	10%(2)	10%(2)
1.0	22%(2)	22%(2)	22%(2)	20%(2)
1.5	36%(2)	36%(2)	36%(2)	32%(2)
2.0	58%(3)	58%(3)	58%(3)	52%(3)
2.5	36%(3)	36%(3)	36%(3)	34%(4)

The success rate of BIC using a frequentist approach was 46%(3).

In the sensitivity analysis where a random intercept and slope model was fitted to data with only random intercept, choosing *α* = 2 led to good results (see S6 Table), whereas in the analysis with a random intercept model, values of *α* lower than 1 were necessary to prevent overestimation of the number of latent classes. These results support setting the value of *α* slightly lower than *d*/2, where *d* is the number of class-specific parameters of the model that is fitted to the data.

### Simulation study A and B: conclusions

Simulation study A shows how the prior for the class-specific parameters and the Dirichlet prior for the class proportion interact to affect the selection of the correct number of latent class models. Using a hierarchical prior (i.e. a normal-gamma prior) for the class-specific means and variances, values for the Dirichlet hyperparameter *α* in the range 0.05–0.10 lead to acceptable results with both moderate or high separation between classes. Higher values for *α* may lead to an overestimation of the number of latent classes, even if *α* remains well below the threshold value *d*/2 that was given in the proof of Rousseau and Mengersen [4]. For *α* < 0.05 a good performance is observed in the high separation scenario, but the number of classes is underestimated in scenarios with a moderate or low amount of separation. This underestimation of the number of latent classes with a low Dirichlet hyperparameter was not observed in a previous simulation study, however that study simulated only data sets with well separated latent classes [16].

With a vague prior for the class-specific means and variances, a perfect performance of the R&M criterion is observed in well separated data sets, irrespective of the value of *α*. An underestimation of the number of classes is observed in the scenarios with a low or moderate separation, especially with low values for *α*. Setting *α* to a higher value, while still ensuring that *α* < *d*/2, led to a considerable improvement in the selection of the number of latent classes in these scenarios. In additional simulations (results not shown), we confirmed that setting *α* to a value above the threshold (i.e. to *α* > *d*/2) results in an overestimation of the number of latent classes, as was predicted by the proof in Rousseau and Mengersen [4].

Using the normal-gamma prior, the performance of the R&M criterion seems quite sensitive to the value of *α*. In addition, the optimal value of *α* (i.e. that leads to highest probability of choosing the correct number of classes) depends on the separation between classes and the true number of classes, which are typically not known in practice (see S7 Table). In contrast the performance of the R&M criterion with a vague prior seems much more stable, as long as the value of *α* is close to but below the threshold of *d*/2. Of the 4 possible values for the threshold to determine whether a class is empty (i.e. *ψ* in Eq 5), we found the best performance using a value of 0.01 in the scenario with a moderate separation (see S8 Table). In the other scenarios there was no clear difference between the possible values of *ψ*. Therefore setting *ψ* = between 0.02 and 0.05 seems reasonable, and a value in this range should allow for the detection of relatively small classes containing a few percent of the population.

Compared to alternative criteria for selecting the number of latent classes, the performance of the R&M criterion was good. The performance of BIC was generally inferior to that of the R&M criterion, especially in data sets with many latent classes and data sets with moderate or low separation. The performance of DIC_3_, DIC_4_ and RJMCMC depends on the value of *α*. Although in some scenarios specific values of *α* seem to lead to a good performance for these criteria, there is no value of *α* that leads a good performance across all scenarios.

Simulation study B confirms the conclusions of simulation study A. It shows that the R&M criterion can also be implemented in a more complex and realistic setting such as a growth mixture model for longitudinal data. The R&M criterion using vague priors for the class-specific parameters and *α* smaller than but close to *d*/2 (e.g. between 0.8 and 0.9 *d*/2) yielded the best results, and outperformed BIC. However, the results were generally less good in Scenario B3, which has a more complex structure with random intercept and slope.

## Hemoglobin longitudinal data

In this section, we apply the R&M criterion to a finite mixture model for hemoglobin (Hb) values of blood donors. Our motivating application is the trajectory of Hb values of blood donors over successive donations. Blood donors experience a temporary reduction in their Hb value after donation. Therefore, a minimum 8 week interval between two donations is set by the blood bank, to allow the donor’s Hb value to recover to its pre-donation level. However, this interval seems to be too short since on average there is a declining trajectory in the Hb values for blood donors who donate regularly [31, 32]. Therefore, a considerable proportion of prospective blood donors are temporarily deferred from donation each year due to low Hb values [33]. A Hb value of 8.4 mmol/l (135 g/l) and 7.8 mmol/l (125 g/l) for men and women, respectively, is widely accepted as the lower cut-off value of eligibility for donation to protect donors from anemia [34]. The previous studies showed that some individuals have a fast recovery, which results in a relatively stable trajectory, whereas others have a slow recovery that yields a declining trajectory in their Hb values [35, 36].

Here, we use a data set of longitudinally observed Hb values from 1 January 2005 to 31 December 2012 collected by Sanquin Blood Supply in the Netherlands. This data set is based on a self-administered questionnaire study aimed at gaining insight into characteristics and motivation of the Dutch donor population [37]. Here we randomly selected 200 new registered male blood donors who have at least 5 visits to blood bank. These data are part of the Donor InSight study, for more details see [37]. The Donor InSight study was approved by the Medical Ethical Committee Arnhem-Nijmegen in the Netherlands, and all participants gave their written informed consent. These data are available in the Supporting Information files (see S1 File).

A mixed-effects model with random intercept and slope may be able to capture the heterogeneity between individuals in these data. However previous studies suggested that describing the total donor population using a single trajectory may oversimplify the complex growth patterns of this population [35, 36]. Therefore, a growth mixture modeling approach, which accounts for different subgroups of donors, seems to be a more appropriate method for capturing differences in Hb trajectories between donors [35, 36]. Here we implemented the R&M criterion with vague priors for the parameters. Different cut-offs (i.e. 0, 0.01, 0.02, and 0.05) were used to define a class to be empty.

Several factors are known to be associated with Hb and hence may be used as predictors, i.e., sex [38], season [39], age [38]. Here we model Hb trajectory based on number of donations in last two years (NODY2), the season donation took place (a binary value for cold = 1 and warm seasons = 0), time since previous donation (TSPD), and age of donor (years) at first visit. The class-specific parameters are the intercept and the effect of NODY2. The aim of the model is to assign each donor to one of *j* groups in such a way that donors with similar Hb trajectories are in the same group, and that the groups are most different from each other in terms of the Hb trajectory.

The growth mixture model for the trajectory of Hb levels of blood donors who belong to latent class *j* can be expressed as:

$$\begin{array}{c} {\text{Hb}}_{it|j}=\theta_{j0}+b_{ij0}+\gamma_{1}{\text{Age}}_{i}+\gamma_{2}{\text{Season}}_{it}+\gamma_{3}{\text{TSPD}}_{it}+\left(\theta_{j1}+b_{ij1}\right){\text{NODY2}}_{it}+\epsilon_{it}, \end{array}$$

where Hb_*it*|*j*_ is the predicted Hb level at the *t*th observation of the *i*th individual, given that this individual is in latent class *j*. *θ*_*j*0_ and *θ*_*j*1_ are the fixed intercept and slope (coefficients of NODY2) of latent class *j*. *b*_*ij*0_ and *b*_*ij*1_ are the random intercept and slope of latent class *j* that are assumed to be bivariate normally distributed with mean zero and a class-specific variance-covariance structure. The residuals *ϵ*_*it*_ are assumed to be normally distributed, and independent of the random effects.

### Prior specification

The priors for the model parameters were chosen as follows. Vague priors were specified for both the class-specific parameters *θ*’s and the non-class-specific parameters *γ*’s, i.e. *N*(0, 10^3^). An Γ^−1^(10^−3^, 10^−3^) was specified for the variance of the residuals. An Inv-Wishart(R, df) distribution was specified for the variance-covariance structure of the random intercept and random slope. We set the degrees of freedom, df, to 3 and the scale parameter matrix, R, to a diagonal matrix with small values, i.e. 10^−3^ [30]. Since the number of class-specific parameters *d* is 5, for the class membership probability a Dirichlet distribution with different values for alpha (i.e., 1.0, 1.5, 2.0, and 2.5) was specified for the mixing proportions.

**Fig 3:**
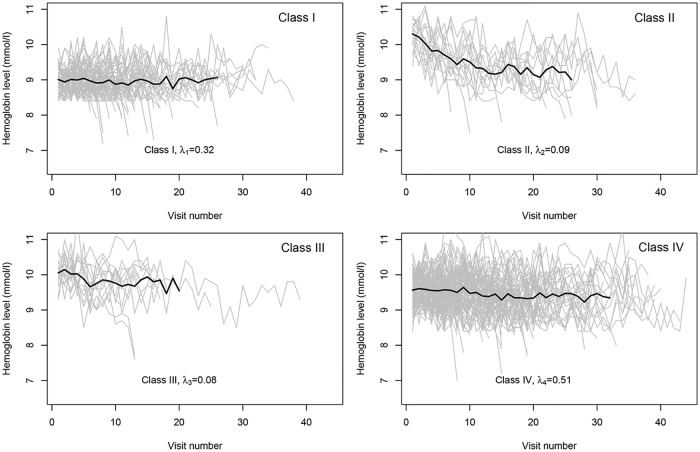
Hb profiles for four different classes.

To analyze these data, we chose the results with *α* = 2, in view of the results of Scenario B3. Therefore, donors can be assigned to four different classes (see Table 6). Based on the highest posterior probability, individuals were assigned to the latent classes after solving the label switching problem using the method suggested by Stephens [40]. This method was implemented in the “label.switching” package in R [41]. The profiles of these different classes are displayed in Fig 3. This figure shows how trajectories of Hb values for blood donors are different. A group of donors have a low initial Hb value but relatively stable trajectory (Class I), donors in Class II have a very high initial Hb value and a very sharply declining trajectory. Donors in Class III have a high initial Hb value and a moderately declining trajectory, donors in Class IV have moderate initial Hb value and relatively stable trajectory. The results of this study regarding the number of latent classes and the interpretation of each class are supported by a previous study [35].

**Table 6:** Number of latent classes in Hb data for different *α* and different cut-offs (*ψ*).

*α*	${\text{R\&M}}_{0}^{\text{NI}}$	${\text{R\&M}}_{0.01}^{\text{NI}}$	${\text{R\&M}}_{0.02}^{\text{NI}}$	${\text{R\&M}}_{0.05}^{\text{NI}}$
0.5	1	1	1	1
1.0	1	1	1	1
1.5	2	2	2	2
2.0	4	4	4	3
2.5	4	4	4	3

BIC using a frequentist approach found 2 classes.

**Fig 4:**
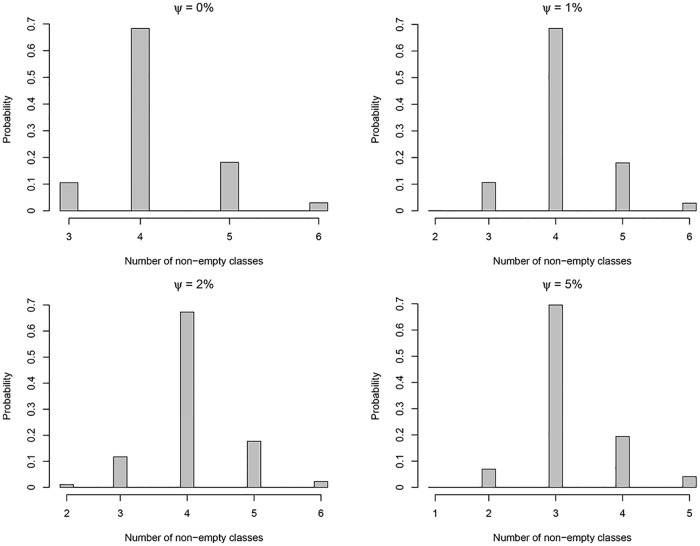
Posterior distribution of non-empty classes (K) for different cut-offs (*ψ*).

Fig 4 shows the posterior distribution of the number of non-empty classes (K) for different cut-offs (*ψ*) using 50,000 MCMC iterations when *α* = 2. This figure shows how the posterior mode of the number of nonempty classes may be affected by changing the *ψ*.

## Discussion

The results of the simulation studies showed that the R&M criterion has a high probability of estimating the correct number of latent classes, provided that the priors on the proportions and the class-specific parameters are chosen carefully. Despite the simplicity of this criterion, it performs at least as good as alternative selection criteria for the number of latent classes. The application of the R&M criterion to longitudinal data of blood donors further illustrated the practical usefulness of this method.

An important advantage of the R&M criterion is that this approach is straightforward to implement, using MCMC sampling for a mixture model with a large number of latent classes. The number of nonempty latent classes (i.e. classes with a proportion larger than the predefined cutoff value) is then an automatic byproduct of the MCMC sampler. Therefore, this criterion is easily implemented in standard Bayesian software such as WinBugs and JAGS, even for complex statistical models such as latent class mixed-effects models and multivariate mixture models. A further advantage of the R&M criterion is that it is not affected by label switching. Despite the fact that the R&M criterion is relatively easy to implement, this criterion seems to perform better than alternative criteria at estimating the true number of classes. Although only a limited set of statistical models was considered in the simulations, these results suggest that the R&M criterion works well and may be considered for practical use in Bayesian finite mixture models.

A strength of this study is that it is one of the first studies to compare different criteria for selecting the number of latent classes in a Bayesian setting. Although the R&M criterion has been implemented in simulated data previously [16], our study adds important insight into how this criterion should be implemented, based on a more elaborate simulation study with several scenarios. In a previous simulation study, it was shown that using a sufficiently low value of *α* (e.g. *α* < 0.001) prevents overfitting of the number of latent classes, and that using higher values of *α*, with *α* < *d*/2 can lead to overfitting [16]. In that study, no underestimation of the number of latent classes was observed. In our simulation study we observed that with a slightly lower amount of separation between classes than in the previous study, underestimation of the number of classes often occurs, especially with low values of *α*. This shows that the value of *α* should be chosen to provide a trade-off between the probability of overfitting and the probability of underfitting the number of latent classes. Unfortunately, no theoretical result is available on how the value of alpha affects the posterior distribution of the class sizes of classes that are not overfitted. Furthermore we observed that if vague priors were used for the class-specific parameters, overfitting of the number of latent classes does not seem to occur, provided that *α* < *d*/2.

Rousseau and Mengersen [4] proved that the class proportions converge to 0, not that they should be 0 with any data set of finite size. We therefore used different cut-offs for the proportions in a class to define a class to be empty. Using a cut-off of 0 may be sensitive to outlying values in the data and did not perform well in the simulation studies (see S4 Table). In most applications, a cut-off of between 0.02 and 0.05 should be sufficient to make the criterion robust to outlying values, while being small enough to avoid the exclusion of real segments in the population. Although choosing the value of the cut-off in the range 0.02–0.05 is supported by results of the simulation studies, there are situations in which lower or higher values of the cut-off may be warranted. First, the interest of finding classes with small proportion may depend on the application and the research questions, and the value of the cut-off may be adapted accordingly. Second, in some applications there can be relevant prior information regarding the class sizes, e.g. if one suspects that there may be classes containing 1% of the population, the cut-off should be set lower than 0.01. Finally, due to the asymptotic nature of the result of R&M, larger sample sizes would generally warrant lower values for the cut-off. However, it should be noted that the rate of convergence proven by R&M is relatively slow, and values of the cut-off between 0.02 and 0.05 seem to be realistic for a wide range of sample sizes. In case of sample sizes much larger than used in our simulation study, the possibility of lower values of the cut-off may be considered. Therefore, in practice the value of the cut-off is to some extent a subjective decision to be made by the researcher, guided by prior knowledge and the level of interest in small subgroups.

Based on the results of the simulation studies, as discussed above, combined with the results of the blood donor data set, we give the following recommendations:

- We recommend to consider the R&M criterion to choose the number of latent classes in Bayesian finite mixture models. This criterion is easy to implement in practice, and its performance compares favorably with alternative criteria.
- To implement the criterion, one should first estimate a mixture model with a large number of classes (e.g. 10 classes), so that some classes will be overfitted.
- The number of classes in the final finite mixture model is then chosen as the posterior mode of the number of classes with a proportion larger than the predefined cut-off, which we recommend to set between 0.02–0.05. Lower values of the cut-off should be used if the researcher is specifically interested in the classes with small proportions in the population.
- It seems best to use vague priors for the class-specific parameters, and the use of hierarchical priors such as the normal-gamma prior is not recommended.
- The class proportions should be given a Dirichlet prior with *α* lower than d/2, i.e. the number of class-specific parameters divided by 2. A value of *α* slightly lower than d/2 (e.g. between 0.8 and 0.9 *d*/2) seems to yield the best results.

A limitation of this study is that only finite mixtures of Gaussian distributions and growth mixtures models were considered in the simulation study. Although the results of the simulation study were similar in these two types of models, it is not certain that the performance of the R&M will be similar in other types of models. Due to the large computation time associated with simulation studies in a Bayesian setting, it was not feasible to consider additional statistical models. Another limitation is that only predefined settings were evaluated for the priors of both class-specific parameters and the class proportions. It is possible that intermediate values of *α* or *ψ*, or also other priors not considered here would lead to a better performance. We further did not consider alternatives to the normal-gamma prior and the vague prior for the class-specific parameters.

### Conclusion

If appropriate priors are used for both the class-specific parameters and the class proportions, it seems possible to effectively estimate the number of latent classes in a Bayesian finite mixture model using the R&M criterion. This criterion compares favorably to alternative model selection criteria for the number of latent classes in terms of both performance and ease of implementation.

## Supporting Information

S1 Table A heterogeneous population with different clusters (*K* = 1, …, 6). *μ*_*j*_ = *j* and *σ*_*j*_ = 0.25, (*j* = 1, …, 6), and (*K*_max_ = 10). Percentage of data sets in which the true number of clusters was found, with the mode of the estimated number of classes in parentheses. A vague prior was used for the class-specific parameters.
(PDF)

S2 Table A heterogeneous population with different clusters (*K* = 1, …, 6). *μ*_*j*_ = *j* and *σ*_*j*_ = 0.25, (*j* = 1, …, 6), and (*K*_max_ = 10). Percentage of data sets in which the true number of clusters was found, with the mode of the estimated number of classes in parentheses. A normal-gamma prior was used for the class-specific parameters.
(PDF)

S3 Table The results of a sensitivity analysis for two different sample sizes i.e., n = 100 and n = 1000. Theses analyses are based on the Scenario A2. Percentage of data sets in which the true number of clusters was found, with the mode of the estimated number of classes in parentheses. A vague prior was used for the class-specific parameters.
(PDF)

S4 Table The results of a sensitivity analysis for the outlying values. This analysis is based the Scenario A1, where two extreme values added at each tail (n = 200). Percentage of data sets in which the true number of clusters was found, with the mode of the estimated number of classes in parentheses. A vague prior was used for the class-specific parameters.
(PDF)

S5 Table The results of Scenario B1. Percentage of data sets in which the true number of clusters was found, with the mode of the estimated number of classes in parentheses.
(PDF)

S6 Table The results of a sensitivity analysis for fitting a more flexible model to the generated data. This analysis is based the Scenario B2 where the most flexible model (a random intercept and slope model) is fitted to data to find the true number of classes. Percentage of data sets in which the true number of clusters was found, with the mode of the estimated number of classes in parentheses. A vague prior was used for the class-specific parameters.
(PDF)

S7 Table A heterogeneous population with different clusters (*K* = 1, …, 6). *μ*_*j*_ = *j* and *σ*_*j*_ = 0.40, (*j* = 1, …, 6), and (*K*_max_ = 10). Percentage of data sets in which the true number of clusters was found, with the mode of the estimated number of classes in parentheses. A normal-gamma prior was used for the class-specific parameters.
(PDF)

S8 Table A heterogeneous population with different clusters (*K* = 1, …, 6). *μ*_*j*_ = *j* and *σ*_*j*_ = 0.40, (*j* = 1, …, 6), and (*K*_max_ = 10). Percentage of data sets in which the true number of clusters was found, with the mode of the estimated number of classes in parentheses. A vague prior was used for the class-specific parameters.
(PDF)

S1 Fig Bugs/Jags codes to implement a univariate Gaussian mixture model with R&M criterion to find the true number of latent classes in Scenario A (R&M^NI^). (TIF)

S2 Fig Bugs/Jags codes to implement a latent class mixed-effects model with R&M criterion to find the true number of latent classes in Scenario B (R&M^NI^). (TIF)

S1 File Hemoglobin longitudinal data. (TXT)
