# Supplementary material for: Comparison of Criteria for Choosing the Number of Classes in Bayesian Finite Mixture Models
Source: PLoS One. 2017 Jan 12;12(1):e0168838. doi: 10.1371/journal.pone.0168838 (PMC5231325; doi:10.1371/journal.pone.0168838)
Supplement: S1 Table — Percentage of data sets in which the true number of clusters was found, with the mode of the estimated number of classes in parentheses. A vague prior was used for the class-specific parameters. (PDF) [file pone.0168838.s001.pdf]

α	Cut-off	$k=1$	$k=2$	$k=3$	$k=4$	$k=5$	$k=6$
0.00001	R&M$_0^{\mathrm{NI}}$	100%(1)	100%(2)	95%(3)	70%(4)	40%(4)	10%(4)
	R&M$_{0.01}^{\mathrm{NI}}$	100%(1)	100%(2)	95%(3)	70%(4)	40%(4)	10%(4)
	R&M$_{0.02}^{\mathrm{NI}}$	100%(1)	100%(2)	95%(3)	70%(4)	40%(4)	10%(4)
	R&M$_{0.05}^{\mathrm{NI}}$	100%(1)	100%(2)	95%(3)	70%(4)	40%(4)	10%(4)
0.001	R&M$_0^{\mathrm{NI}}$	100%(1)	100%(2)	95%(3)	70%(4)	30%(4)	0%(4)
	R&M$_{0.01}^{\mathrm{NI}}$	100%(1)	100%(2)	95%(3)	70%(4)	30%(4)	0%(4)
	R&M$_{0.02}^{\mathrm{NI}}$	100%(1)	100%(2)	95%(3)	70%(4)	30%(4)	0%(4)
	R&M$_{0.05}^{\mathrm{NI}}$	100%(1)	100%(2)	95%(3)	70%(4)	30%(4)	0%(4)
0.01	R&M$_0^{\mathrm{NI}}$	100%(1)	100%(2)	95%(3)	75%(4)	55%(5)	5%(4)
	R&M$_{0.01}^{\mathrm{NI}}$	100%(1)	100%(2)	95%(3)	75%(4)	55%(5)	5%(4)
	R&M$_{0.02}^{\mathrm{NI}}$	100%(1)	100%(2)	95%(3)	75%(4)	55%(5)	5%(4)
	R&M$_{0.05}^{\mathrm{NI}}$	100%(1)	100%(2)	95%(3)	75%(4)	55%(5)	5%(4)
0.05	R&M$_0^{\mathrm{NI}}$	100%(1)	100%(2)	100%(3)	75%(4)	60%(5)	35%(5)
	R&M$_{0.01}^{\mathrm{NI}}$	100%(1)	100%(2)	100%(3)	75%(4)	60%(5)	35%(5)
	R&M$_{0.02}^{\mathrm{NI}}$	100%(1)	100%(2)	100%(3)	75%(4)	60%(5)	35%(5)
	R&M$_{0.05}^{\mathrm{NI}}$	100%(1)	100%(2)	100%(3)	75%(4)	60%(5)	35%(5)
0.1	R&M$_0^{\mathrm{NI}}$	100%(1)	100%(2)	100%(3)	80%(4)	80%(5)	55%(6)
	R&M$_{0.01}^{\mathrm{NI}}$	100%(1)	100%(2)	100%(3)	80%(4)	80%(5)	50%(6)
	R&M$_{0.02}^{\mathrm{NI}}$	100%(1)	100%(2)	100%(3)	80%(4)	80%(5)	50%(6)
	R&M$_{0.05}^{\mathrm{NI}}$	100%(1)	100%(2)	100%(3)	80%(4)	80%(5)	45%(5)
0.3	R&M$_0^{\mathrm{NI}}$	100%(1)	100%(2)	100%(3)	100%(4)	100%(5)	95%(6)
	R&M$_{0.01}^{\mathrm{NI}}$	100%(1)	100%(2)	100%(3)	100%(4)	100%(5)	90%(6)
	R&M$_{0.02}^{\mathrm{NI}}$	100%(1)	100%(2)	100%(3)	100%(4)	100%(5)	90%(6)
	R&M$_{0.05}^{\mathrm{NI}}$	100%(1)	100%(2)	100%(3)	100%(4)	100%(5)	90%(6)
0.5	R&M$_0^{\mathrm{NI}}$	100%(1)	100%(2)	100%(3)	100%(4)	100%(5)	85%(6)
	R&M$_{0.01}^{\mathrm{NI}}$	100%(1)	100%(2)	100%(3)	100%(4)	100%(5)	100%(6)
	R&M$_{0.02}^{\mathrm{NI}}$	100%(1)	100%(2)	100%(3)	100%(4)	100%(5)	100%(6)
	R&M$_{0.05}^{\mathrm{NI}}$	100%(1)	100%(2)	100%(3)	100%(4)	100%(5)	100%(6)
0.9	R&M$_0^{\mathrm{NI}}$	95%(1)	95%(2)	45%(3)	0%(5)	0%(6)	0%(7)
	R&M$_{0.01}^{\mathrm{NI}}$	100%(1)	100%(2)	100%(3)	70%(4)	25%(6)	10%(7)
	R&M$_{0.02}^{\mathrm{NI}}$	100%(1)	100%(2)	100%(3)	85%(4)	60%(5)	30%(7)
	R&M$_{0.05}^{\mathrm{NI}}$	100%(1)	100%(2)	100%(3)	100%(4)	95%(5)	90%(6)
frequentist	BIC	100%(1)	100%(2)	100%(3)	65%(4)	45%(5)	15%(7)
