# Supplementary material for: Comparison of Criteria for Choosing the Number of Classes in Bayesian Finite Mixture Models
Source: PLoS One. 2017 Jan 12;12(1):e0168838. doi: 10.1371/journal.pone.0168838 (PMC5231325; doi:10.1371/journal.pone.0168838)
Supplement: S2 Table — Percentage of data sets in which the true number of clusters was found, with the mode of the estimated number of classes in parentheses. A normal-gamma prior was used for the class-specific parameters. (PDF) [file pone.0168838.s002.pdf]

α	Cut-off	$k=1$	$k=2$	$k=3$	$k=4$	$k=5$	$k=6$
0.00001	R&M$_0^{NG}$	100%(1)	100%(2)	100%(3)	100%(4)	100%(5)	70%(6)
	R&M$_{0.01}^{NG}$	100%(1)	100%(2)	100%(3)	100%(4)	100%(5)	70%(6)
	R&M$_{0.02}^{NG}$	100%(1)	100%(2)	100%(3)	100%(4)	100%(5)	70%(6)
	R&M$_{0.05}^{NG}$	100%(1)	100%(2)	100%(3)	100%(4)	100%(5)	70%(6)
0.001	R&M$_0^{NG}$	100%(1)	100%(2)	100%(3)	100%(4)	100%(5)	100%(6)
	R&M$_{0.01}^{NG}$	100%(1)	100%(2)	100%(3)	100%(4)	100%(5)	100%(6)
	R&M$_{0.02}^{NG}$	100%(1)	100%(2)	100%(3)	100%(4)	100%(5)	100%(6)
	R&M$_{0.05}^{NG}$	100%(1)	100%(2)	100%(3)	100%(4)	100%(5)	100%(6)
0.01	R&M$_0^{NG}$	95%(1)	100%(2)	100%(3)	100%(4)	100%(5)	100%(6)
	R&M$_{0.01}^{NG}$	100%(1)	100%(2)	100%(3)	100%(4)	100%(5)	100%(6)
	R&M$_{0.02}^{NG}$	100%(1)	100%(2)	100%(3)	100%(4)	100%(5)	100%(6)
	R&M$_{0.05}^{NG}$	100%(1)	100%(2)	100%(3)	100%(4)	100%(5)	100%(6)
0.05	R&M$_0^{NG}$	30%(2)	5%(3)	0%(3)	5%(5)	40%(6)	80%(6)
	R&M$_{0.01}^{NG}$	55%(1)	85%(2)	95%(3)	100%(4)	95%(5)	100%(6)
	R&M$_{0.02}^{NG}$	60%(1)	95%(2)	100%(3)	100%(4)	95%(5)	100%(6)
	R&M$_{0.05}^{NG}$	80%(1)	95%(2)	100%(3)	100%(4)	100%(5)	100%(6)
0.1	R&M$_0^{NG}$	0%(3)	0%(4)	0%(5)	0%(6)	0%(6)	0%(7)
	R&M$_{0.01}^{NG}$	0%(3)	0%(3)	0%(4)	0%(5)	15%(6)	80%(6)
	R&M$_{0.02}^{NG}$	0%(2)	0%(3)	10%(4)	20%(5)	70%(5)	100%(6)
	R&M$_{0.05}^{NG}$	10%(2)	45%(3)	80%(3)	100%(4)	100%(5)	100%(6)
0.3	R&M$_0^{NG}$	0%(7)	0%(7)	0%(8)	0%(8)	0%(9)	0%(9)
	R&M$_{0.01}^{NG}$	0%(6)	0%(6)	0%(6)	0%(7)	0%(7)	0%(8)
	R&M$_{0.02}^{NG}$	0%(5)	0%(5)	0%(6)	0%(6)	0%(7)	0%(7)
	R&M$_{0.05}^{NG}$	0%(4)	0%(4)	0%(4)	0%(5)	0%(6)	75%(6)
0.5	R&M$_0^{NG}$	0%(8)	0%(9)	0%(9)	0%(9)	0%(10)	0%(10)
	R&M$_{0.01}^{NG}$	0%(7)	0%(7)	0%(7)	0%(8)	0%(8)	0%(9)
	R&M$_{0.02}^{NG}$	0%(6)	0%(6)	0%(7)	0%(7)	0%(8)	0%(8)
	R&M$_{0.05}^{NG}$	0%(5)	0%(5)	0%(5)	0%(6)	0%(6)	5%(7)
0.9	R&M$_0^{NG}$	0%(9)	0%(10)	0%(10)	0%(10)	0%(10)	0%(10)
	R&M$_{0.01}^{NG}$	0%(8)	0%(9)	0%(9)	0%(9)	0%(9)	0%(9)
	R&M$_{0.02}^{NG}$	0%(8)	0%(8)	0%(8)	0%(8)	0%(8)	0%(9)
	R&M$_{0.05}^{NG}$	0%(6)	0%(6)	0%(6)	0%(6)	0%(7)	0%(7)
frequentist	BIC	100%(1)	100%(2)	100%(3)	65%(4)	45%(5)	15%(7)
