# Supplementary material for: Comparison of Criteria for Choosing the Number of Classes in Bayesian Finite Mixture Models
Source: PLoS One. 2017 Jan 12;12(1):e0168838. doi: 10.1371/journal.pone.0168838 (PMC5231325; doi:10.1371/journal.pone.0168838)
Supplement: S3 Table — Theses analyses are based on the Scenario A2. Percentage of data sets in which the true number of clusters was found, with the mode of the estimated number of classes in parentheses. A vague prior was used for the class-specific parameters. (PDF) [file pone.0168838.s003.pdf]

Sample size	α	$R\&M^{NI}_{0}$	$R\&M^{NI}_{0.01}$	$R\&M^{NI}_{0.02}$	$R\&M^{NI}_{0.05}$
#n=100	0.00001	0%(1)	0%(1)	0%(1)	0%(1)
	0.001	0%(1)	0%(1)	0%(1)	0%(1)
	0.01	4%(1)	4%(1)	4%(1)	4%(1)
	0.05	14%(2)	14%(2)	14%(2)	14%(2)
	0.1	16%(2)	16%(2)	16%(2)	16%(2)
	0.3	22%(2)	22%(2)	22%(2)	22%(2)
	0.5	44%(2)	34%(2)	32%(2)	28%(2)
	0.9	96%(3)	92%(3)	84%(3)	76%(3)
#n=1000	0.00001	100%(3)	100%(3)	100%(3)	100%(3)
	0.001	100%(3)	100%(3)	100%(3)	100%(3)
	0.01	100%(3)	100%(3)	100%(3)	100%(3)
	0.05	100%(3)	100%(3)	100%(3)	100%(3)
	0.1	100%(3)	100%(3)	100%(3)	100%(3)
	0.3	100%(3)	100%(3)	100%(3)	100%(3)
	0.5	98%(3)	100%(3)	100%(3)	100%(3)
	0.9	2%(4)	60%(3)	72%(3)	88%(3)
