# Supplementary material for: Comparison of Criteria for Choosing the Number of Classes in Bayesian Finite Mixture Models
Source: PLoS One. 2017 Jan 12;12(1):e0168838. doi: 10.1371/journal.pone.0168838 (PMC5231325; doi:10.1371/journal.pone.0168838)
Supplement: S4 Table — This analysis is based the Scenario A1, where two extreme values added at each tail (n = 200). Percentage of data sets in which the true number of clusters was found, with the mode of the estimated number of classes in parentheses. A vague prior was used for the class-specific parameters. (PDF) [file pone.0168838.s004.pdf]

α	R&M$_0^{\mathrm{NI}}$	R&M$_{0.01}^{\mathrm{NI}}$	R&M$_{0.02}^{\mathrm{NI}}$	R&M$_{0.05}^{\mathrm{NI}}$
0.00001	0%(2)	0%(2)	0%(2)	100%(1)
0.001	0%(2)	0%(2)	0%(2)	100%(1)
0.01	0%(2)	0%(2)	0%(2)	100%(1)
0.05	0%(2)	0%(2)	0%(2)	100%(1)
0.1	0%(2)	0%(2)	0%(2)	100%(1)
0.3	0%(2)	0%(2)	10%(2)	100%(1)
0.5	0%(3)	0%(2)	50%(1)	100%(1)
0.9	0%(3)	0%(2)	60%(1)	100%(1)
