# Supplementary material for: Comparison of Criteria for Choosing the Number of Classes in Bayesian Finite Mixture Models
Source: PLoS One. 2017 Jan 12;12(1):e0168838. doi: 10.1371/journal.pone.0168838 (PMC5231325; doi:10.1371/journal.pone.0168838)
Supplement: S5 Table — Percentage of data sets in which the true number of clusters was found, with the mode of the estimated number of classes in parentheses. (PDF) [file pone.0168838.s005.pdf]

α	R&M$_0^{NI}$	R&M$_{0.01}^{NI}$	R&M$_{0.02}^{NI}$	R&M$_{0.05}^{NI}$
0.00001	100%(1)	100%(1)	100%(1)	100%(1)
0.001	100%(1)	100%(1)	100%(1)	100%(1)
0.01	100%(1)	100%(1)	100%(1)	100%(1)
0.05	100%(1)	100%(1)	100%(1)	100%(1)
0.1	100%(1)	100%(1)	100%(1)	100%(1)
0.3	100%(1)	100%(1)	100%(1)	100%(1)
0.5	100%(1)	100%(1)	100%(1)	100%(1)
1.0	100%(1)	100%(1)	100%(1)	100%(1)
1.5	100%(1)	100%(1)	100%(1)	100%(1)
2.0	98%(1)	98%(1)	98%(1)	98%(1)

The success rate of BIC using a frequentist approach was 100%.
