# Supplementary material for: Comparison of Criteria for Choosing the Number of Classes in Bayesian Finite Mixture Models
Source: PLoS One. 2017 Jan 12;12(1):e0168838. doi: 10.1371/journal.pone.0168838 (PMC5231325; doi:10.1371/journal.pone.0168838)
Supplement: S6 Table — This analysis is based the Scenario B2 where the most flexible model (a random intercept and slope model) is fitted to data to find the true number of classes. Percentage of data sets in which the true number of clusters was found, with the mode of the estimated number of classes in parentheses. A vague prior was used for the class-specific parameters. (PDF) [file pone.0168838.s006.pdf]

α	$R\&M_0^{NI}$	$R\&M_{0.01}^{NI}$	$R\&M_{0.02}^{NI}$	$R\&M_{0.05}^{NI}$
0.00001	20%(2)	20%(2)	20%(2)	20%(2)
0.001	26%(2)	26%(2)	26%(2)	26%(2)
0.01	30%(2)	30%(2)	30%(2)	30%(2)
0.05	32%(2)	32%(2)	32%(2)	32%(2)
0.1	30%(2)	30%(2)	30%(2)	30%(2)
0.3	38%(2)	38%(2)	38%(2)	38%(2)
0.5	40%(2)	40%(2)	40%(2)	40%(2)
1.0	58%(3)	58%(3)	58%(3)	58%(3)
1.5	66%(3)	66%(3)	66%(3)	66%(3)
2.0	84%(3)	84%(3)	84%(3)	84%(3)
2.5	88%(3)	88%(3)	88%(3)	88%(3)
