# Supplementary material for: Comparison of Criteria for Choosing the Number of Classes in Bayesian Finite Mixture Models
Source: PLoS One. 2017 Jan 12;12(1):e0168838. doi: 10.1371/journal.pone.0168838 (PMC5231325; doi:10.1371/journal.pone.0168838)
Supplement: S7 Table — Percentage of data sets in which the true number of clusters was found, with the mode of the estimated number of classes in parentheses. A normal-gamma prior was used for the class-specific parameters. (PDF) [file pone.0168838.s007.pdf]

α	Cut-off	$k=1$	$k=2$	$k=3$	$k=4$	$k=5$	$k=6$
0.00001	$\mathrm{R\&M}_{0}^{\mathrm{NG}}$	100%(1)	15%(1)	0%(2)	0%(2)	0%(2)	0%(3)
	$\mathrm{R\&M}_{0.01}^{\mathrm{NG}}$	100%(1)	15%(1)	0%(2)	0%(2)	0%(2)	0%(3)
	$\mathrm{R\&M}_{0.02}^{\mathrm{NG}}$	100%(1)	15%(1)	0%(2)	0%(2)	0%(2)	0%(3)
	$\mathrm{R\&M}_{0.05}^{\mathrm{NG}}$	100%(1)	15%(1)	0%(2)	0%(2)	0%(2)	0%(3)
0.001	$\mathrm{R\&M}_{0}^{\mathrm{NG}}$	100%(1)	20%(1)	0%(2)	0%(2)	0%(3)	0%(3)
	$\mathrm{R\&M}_{0.01}^{\mathrm{NG}}$	100%(1)	20%(1)	0%(2)	0%(2)	0%(3)	0%(3)
	$\mathrm{R\&M}_{0.02}^{\mathrm{NG}}$	100%(1)	15%(1)	0%(2)	0%(2)	0%(3)	0%(3)
	$\mathrm{R\&M}_{0.05}^{\mathrm{NG}}$	100%(1)	15%(1)	0%(2)	0%(2)	0%(3)	0%(3)
0.01	$\mathrm{R\&M}_{0}^{\mathrm{NG}}$	100%(1)	55%(2)	25%(2)	0%(3)	0%(3)	0%(3)
	$\mathrm{R\&M}_{0.01}^{\mathrm{NG}}$	100%(1)	50%(2)	20%(2)	0%(3)	0%(3)	0%(3)
	$\mathrm{R\&M}_{0.02}^{\mathrm{NG}}$	100%(1)	50%(2)	20%(2)	0%(3)	0%(3)	0%(3)
	$\mathrm{R\&M}_{0.05}^{\mathrm{NG}}$	100%(1)	50%(2)	20%(2)	0%(3)	0%(3)	0%(3)
0.05	$\mathrm{R\&M}_{0}^{\mathrm{NG}}$	30%(2)	40%(3)	75%(3)	85%(4)	20%(4)	0%(4)
	$\mathrm{R\&M}_{0.01}^{\mathrm{NG}}$	65%(1)	85%(2)	75%(3)	10%(3)	0%(3)	0%(4)
	$\mathrm{R\&M}_{0.02}^{\mathrm{NG}}$	70%(1)	95%(2)	60%(3)	5%(3)	0%(3)	0%(4)
	$\mathrm{R\&M}_{0.05}^{\mathrm{NG}}$	80%(1)	90%(2)	40%(2)	0%(3)	0%(3)	0%(4)
0.1	$\mathrm{R\&M}_{0}^{\mathrm{NG}}$	0%(3)	0%(4)	0%(5)	0%(5)	65%(5)	65%(6)
	$\mathrm{R\&M}_{0.01}^{\mathrm{NG}}$	0%(3)	5%(3)	15%(4)	100%(4)	40%(5)	0%(5)
	$\mathrm{R\&M}_{0.02}^{\mathrm{NG}}$	0%(2)	10%(3)	50%(3)	100%(4)	25%(5)	0%(4)
	$\mathrm{R\&M}_{0.05}^{\mathrm{NG}}$	10%(2)	45%(3)	95%(3)	40%(3)	0%(4)	0%(4)
0.3	$\mathrm{R\&M}_{0}^{\mathrm{NG}}$	0%(7)	0%(7)	0%(8)	0%(8)	0%(8)	0%(0)
	$\mathrm{R\&M}_{0.01}^{\mathrm{NG}}$	0%(6)	0%(6)	0%(6)	0%(7)	0%(7)	0%(7)
	$\mathrm{R\&M}_{0.02}^{\mathrm{NG}}$	0%(5)	0%(5)	0%(6)	0%(6)	0%(6)	95%(6)
	$\mathrm{R\&M}_{0.05}^{\mathrm{NG}}$	0%(4)	0%(4)	0%(5)	0%(5)	100%(5)	10%(5)
0.5	$\mathrm{R\&M}_{0}^{\mathrm{NG}}$	0%(8)	0%(9)	0%(9)	0%(9)	0%(9)	0%(10)
	$\mathrm{R\&M}_{0.01}^{\mathrm{NG}}$	0%(7)	0%(7)	0%(8)	0%(8)	0%(8)	0%(8)
	$\mathrm{R\&M}_{0.02}^{\mathrm{NG}}$	0%(6)	0%(6)	0%(7)	0%(7)	0%(7)	0%(7)
	$\mathrm{R\&M}_{0.05}^{\mathrm{NG}}$	0%(5)	0%(5)	0%(5)	0%(6)	0%(6)	100%(6)
0.9	$\mathrm{R\&M}_{0}^{\mathrm{NG}}$	0%(9)	0%(10)	0%(10)	0%(10)	0%(10)	0%(10)
	$\mathrm{R\&M}_{0.01}^{\mathrm{NG}}$	0%(8)	0%(9)	0%(9)	0%(9)	0%(9)	0%(9)
	$\mathrm{R\&M}_{0.02}^{\mathrm{NG}}$	0%(8)	0%(8)	0%(8)	0%(8)	0%(8)	0%(8)
	$\mathrm{R\&M}_{0.05}^{\mathrm{NG}}$	0%(6)	0%(6)	0%(6)	0%(6)	0%(7)	15%(7)
frequentist	BIC	100%(1)	45%(1)	15%(2)	0%(3)	0%(3)	0%(3)
