# Supplementary material for: Comparison of Criteria for Choosing the Number of Classes in Bayesian Finite Mixture Models
Source: PLoS One. 2017 Jan 12;12(1):e0168838. doi: 10.1371/journal.pone.0168838 (PMC5231325; doi:10.1371/journal.pone.0168838)
Supplement: S8 Table — Percentage of data sets in which the true number of clusters was found, with the mode of the estimated number of classes in parentheses. A vague prior was used for the class-specific parameters. (PDF) [file pone.0168838.s008.pdf]

α	Cut-off	$k=1$	$k=2$	$k=3$	$k=4$	$k=5$	$k=6$
0.00001	R&M$_{0}^{\mathrm{NI}}$	100%(1)	5%(1)	0%(2)	0%(2)	0%(3)	0%(3)
	R&M$_{0.01}^{\mathrm{NI}}$	100%(1)	5%(1)	0%(2)	0%(2)	0%(3)	0%(3)
	R&M$_{0.02}^{\mathrm{NI}}$	100%(1)	5%(1)	0%(2)	0%(2)	0%(3)	0%(3)
	R&M$_{0.05}^{\mathrm{NI}}$	100%(1)	5%(1)	0%(2)	0%(2)	0%(3)	0%(3)
0.001	R&M$_{0}^{\mathrm{NI}}$	100%(1)	5%(1)	0%(2)	0%(2)	0%(3)	0%(3)
	R&M$_{0.01}^{\mathrm{NI}}$	100%(1)	5%(1)	0%(2)	0%(2)	0%(3)	0%(3)
	R&M$_{0.02}^{\mathrm{NI}}$	100%(1)	5%(1)	0%(2)	0%(2)	0%(3)	0%(3)
	R&M$_{0.05}^{\mathrm{NI}}$	100%(1)	5%(1)	0%(2)	0%(2)	0%(3)	0%(3)
0.01	R&M$_{0}^{\mathrm{NI}}$	100%(1)	5%(1)	0%(2)	0%(2)	0%(3)	0%(3)
	R&M$_{0.01}^{\mathrm{NI}}$	100%(1)	5%(1)	0%(2)	0%(2)	0%(3)	0%(3)
	R&M$_{0.02}^{\mathrm{NI}}$	100%(1)	5%(1)	0%(2)	0%(2)	0%(3)	0%(3)
	R&M$_{0.05}^{\mathrm{NI}}$	100%(1)	5%(1)	0%(2)	0%(2)	0%(3)	0%(3)
0.05	R&M$_{0}^{\mathrm{NI}}$	100%(1)	5%(1)	0%(2)	0%(2)	0%(3)	0%(3)
	R&M$_{0.01}^{\mathrm{NI}}$	100%(1)	5%(1)	0%(2)	0%(2)	0%(3)	0%(3)
	R&M$_{0.02}^{\mathrm{NI}}$	100%(1)	5%(1)	0%(2)	0%(2)	0%(3)	0%(3)
	R&M$_{0.05}^{\mathrm{NI}}$	100%(1)	5%(1)	0%(2)	0%(2)	0%(3)	0%(3)
0.1	R&M$_{0}^{\mathrm{NI}}$	100%(1)	5%(1)	0%(2)	0%(2)	0%(3)	0%(3)
	R&M$_{0.01}^{\mathrm{NI}}$	100%(1)	5%(1)	0%(2)	0%(2)	0%(3)	0%(3)
	R&M$_{0.02}^{\mathrm{NI}}$	100%(1)	5%(1)	0%(2)	0%(2)	0%(3)	0%(3)
	R&M$_{0.05}^{\mathrm{NI}}$	100%(1)	5%(1)	0%(2)	0%(2)	0%(3)	0%(3)
0.3	R&M$_{0}^{\mathrm{NI}}$	100%(1)	40%(1)	5%(2)	0%(3)	0%(3)	0%(4)
	R&M$_{0.01}^{\mathrm{NI}}$	100%(1)	35%(1)	5%(2)	0%(3)	0%(3)	0%(3)
	R&M$_{0.02}^{\mathrm{NI}}$	100%(1)	35%(1)	5%(2)	0%(3)	0%(3)	0%(3)
	R&M$_{0.05}^{\mathrm{NI}}$	100%(1)	35%(1)	5%(2)	0%(3)	0%(3)	0%(3)
0.5	R&M$_{0}^{\mathrm{NI}}$	90%(1)	55%(2)	25%(3)	5%(3)	0%(4)	0%(5)
	R&M$_{0.01}^{\mathrm{NI}}$	90%(1)	55%(2)	15%(3)	0%(3)	0%(3)	0%(4)
	R&M$_{0.02}^{\mathrm{NI}}$	100%(1)	55%(2)	15%(3)	0%(3)	0%(3)	0%(4)
	R&M$_{0.05}^{\mathrm{NI}}$	100%(1)	55%(2)	15%(3)	0%(3)	0%(3)	0%(4)
0.9	R&M$_{0}^{\mathrm{NI}}$	80%(1)	65%(2)	25%(4)	15%(5)	10%(6)	15%(7)
	R&M$_{0.01}^{\mathrm{NI}}$	95%(1)	95%(2)	95%(3)	95%(4)	95%(5)	95%(6)
	R&M$_{0.02}^{\mathrm{NI}}$	95%(1)	90%(2)	95%(3)	95%(4)	90%(5)	30%(5)
	R&M$_{0.05}^{\mathrm{NI}}$	100%(1)	95%(2)	90%(3)	60%(4)	25%(4)	5%(5)
frequentist	BIC	100%(1)	45%(1)	15%(2)	0%(3)	0%(3)	0%(3)
