# Supplementary figures and images for: Comparison of Criteria for Choosing the Number of Classes in Bayesian Finite Mixture Models
Source: PLoS One. 2017 Jan 12;12(1):e0168838. doi: 10.1371/journal.pone.0168838 (PMC5231325; doi:10.1371/journal.pone.0168838)

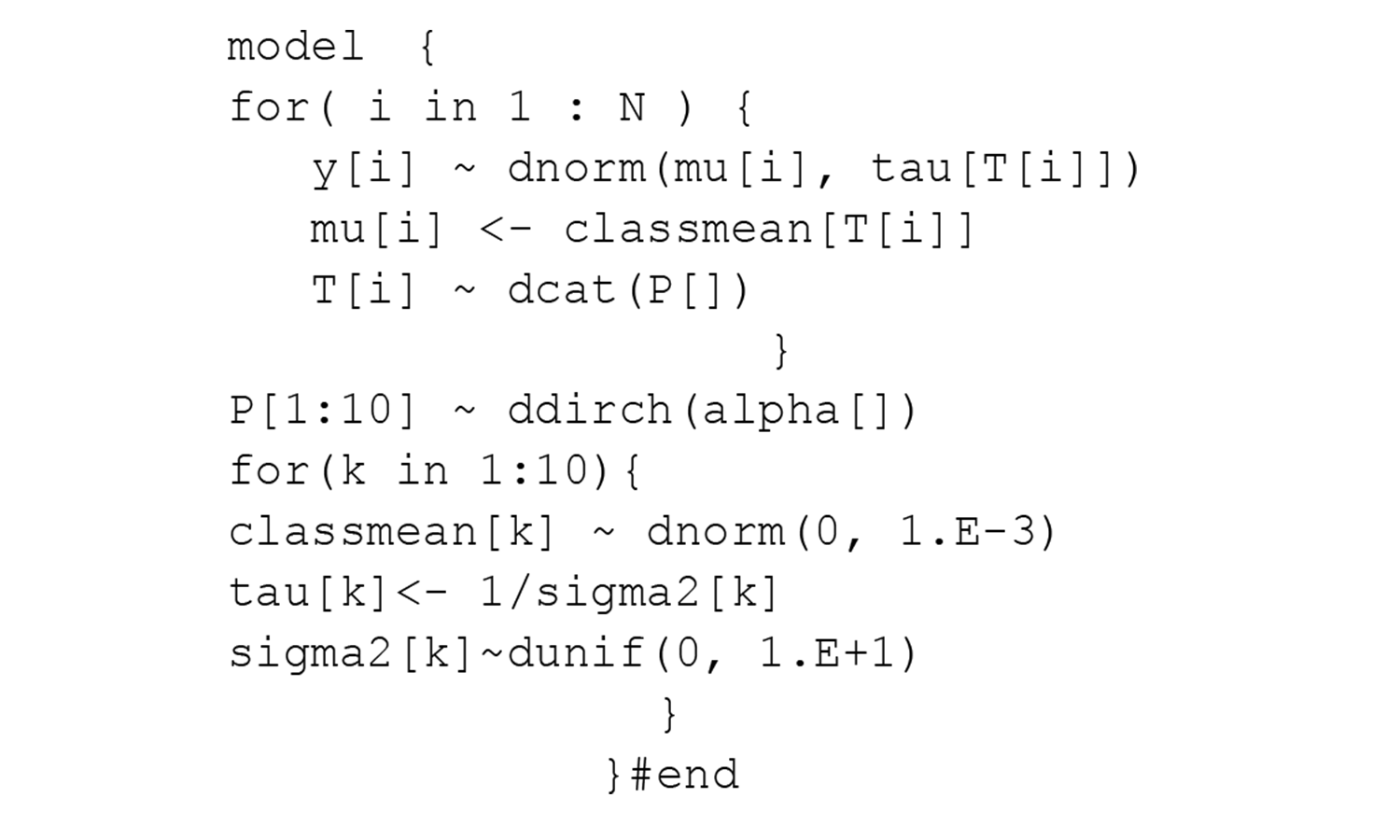

Supplement: S1 Fig — (TIF) [file pone.0168838.s009.tif]

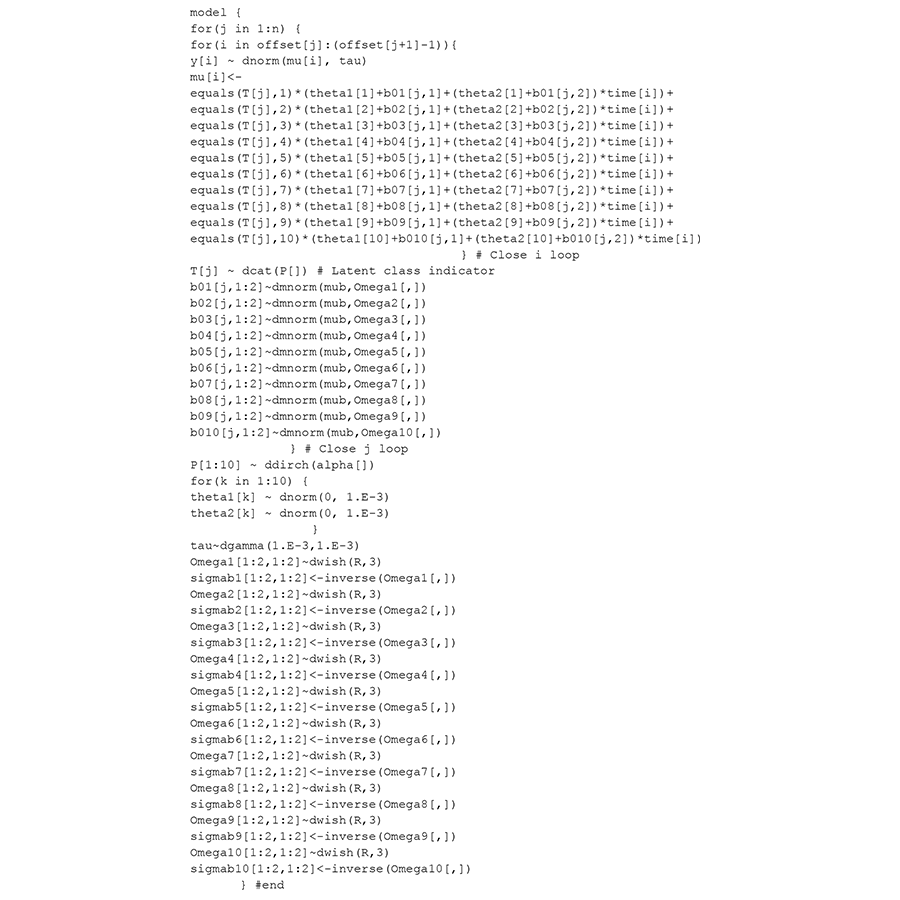

Supplement: S2 Fig — (TIF) [file pone.0168838.s010.tif]
